# Research diagnostic criteria for mild cognitive impairment with Lewy bodies: A systematic review and meta-analysis

**DOI:** 10.1002/alz.13105

**Published:** 2023-04-24

**Authors:** Paul C Donaghy, Claudia Carrarini, Daniel Ferreira, Annegret Habich, Dag Aarsland, Claudio Babiloni, Ece Bayram, Joseph PM Kane, Simon JG Lewis, Andrea Pilotto, Alan J Thomas, Laura Bonanni

**Affiliations:** 1 Translational and Clinical Research Institute, Newcastle University, Newcastle upon Tyne, UK; 2 Department of Neuroscience, Catholic University of Sacred Heart, Rome, Italy; 3 IRCCS San Raffaele Pisana, Rome, Italy; 4 Division of Clinical Geriatrics, Center for Alzheimer Research, Department of Neurobiology, Care Sciences and Society, Karolinska Institutet, Stockholm, Sweden; 5 Department of Radiology, Mayo Clinic, Rochester, Minnesota, USA; 6 University Hospital of Psychiatry and Psychotherapy, University of Bern, Bern, Switzerland; 7 Department of Old Age Psychiatry, Institute of Psychiatry, Psychology & Neuroscience, King’s College London, London, UK; 8 Centre for Age-Related Diseases, Stavanger University Hospital, Stavanger, Norway; 9 Department of Physiology and Pharmacology “Vittorio Erspamer”, Sapienza University of Rome, Rome, Italy; 10 Hospital San Raffaele of Cassino, Cassino, Italy; 11 Parkinson and Other Movement Disorders Center, Department of Neurosciences, University of California San Diego, California, USA; 12 Centre for Public Health, Queen’s University Belfast, Belfast, UK; 13 Brain and Mind Centre, School of Medical Sciences, University of Sydney, Sydney, Australia; 14 Department of Clinical and Experimental Sciences, Neurology Unit, University of Brescia, Brescia, Italy; 15 Department of Medicine and Aging Sciences, University G. d’Annunzio of Chieti-Pescara, Chieti, Italy

**Keywords:** biomarkers, diagnosis, diagnostic criteria, dementia with Lewy bodies, electroencephalography, imaging, Lewy body disease, mild cognitive impairment, symptoms

## Abstract

**Introduction:**

Operationalized research criteria for mild cognitive impairment with Lewy bodies (MCI-LB) were published in 2020. The aim of this systematic review and meta-analysis was to review the evidence for the diagnostic clinical features and biomarkers in MCI-LB set out in the criteria.

**Methods:**

MEDLINE, PubMed, and Embase were searched on 9/28/22 for relevant articles. Articles were included if they presented original data reporting the rates of diagnostic features in MCI-LB.

**Results:**

Fifty-seven articles were included. The meta-analysis supported the inclusion of the current clinical features in the diagnostic criteria. Evidence for striatal dopaminergic imaging and meta-iodobenzylguanidine cardiac scintigraphy, though limited, supports their inclusion. Quantitative electroencephalogram (EEG) and fluorodeoxyglucose positron emission tomography (PET) show promise as diagnostic biomarkers.

**Discussion:**

The available evidence largely supports the current diagnostic criteria for MCI-LB. Further evidence will help refine the diagnostic criteria and understand how best to apply them in clinical practice and research.

## 1 | BACKGROUND

The diagnosis of mild cognitive impairment (MCI) has become a routine part of clinical care in aging and dementia. In memory clinics, one in five attendees receives a diagnosis of MCI.^1^ In research, clinical trials are moving toward treatment in MCI and earlier disease stages, facilitated by criteria to identify specific causes of MCI, such as MCI due to Alzheimer’s disease (MCI-AD).^2^ In this context, research criteria for the diagnosis of prodromal dementia with Lewy bodies (DLB) were published in 2020.^3^ These included operationalized criteria for the diagnosis of MCI with Lewy bodies (MCI-LB). The criteria identified core clinical features (cognitive fluctuations, recurrent visual hallucinations, rapid eye movement [REM], sleep behavior disorder [RBD], and parkinsonism) and proposed biomarkers (abnormal striatal dopaminergic imaging, abnormal meta-iodobenzylguanidine [MIBG] cardiac scintigraphy, and polysomnography confirmation of REM sleep without atonia). These core clinical features and proposed biomarkers are included in the diagnostic algorithm (see Table 1 for details). The criteria also include supportive clinical features and potential biomarkers, which are not included in the diagnostic algorithm, but are thought to be consistent with underlying Lewy body (LB) disease.

The criteria were developed based on expert consensus and the authors advised that they should be validated in prospective cohorts before entering routine clinical use. Since the criteria were agreed, there has been a significant increase in publications relating to MCI-LB. The Alzheimer’s Association International Society to Advance Alzheimer’s Research and Treatment (ISTAART) Lewy Body Dementias Professional Interest Area (PIA) Prodromal DLB Working Group agreed that a review of all evidence for the MCI-LB criteria would be of great benefit to the field.

The objective of this article was to systematically review the evidence for the prevalence rates of core clinical features, supportive clinical features, proposed biomarkers, and potential biomarkers in MCI-LB, including meta-analytical estimations of prevalence rates. Where possible, we compared these with the prevalence of these features in other forms of MCI, such as MCI-AD and stable MCI.

## 2 | METHODS

### 2.1 | Eligibility criteria

We followed the PRISMA guidelines for systematic reviews and meta-analyses.^4^

Inclusion criteria: original data investigating the presence of one or more of the diagnostic features in MCI-LB. The presence of diagnostic features in MCI-LB could be demonstrated by (1) data from the MCI phase in cases of autopsy-confirmed Lewy body (LB) disease (*post mortem* studies); (2) data from the MCI phase in participants diagnosed with clinical DLB at later follow-up assessments; (3) data from cohorts of clinically diagnosed MCI-LB, defined using recognized criteria (e.g., McKeith 2020 or previous McKeith DLB criteria applied to MCI cases). For prevalence rates of core clinical features, only data from articles demonstrating MCI-LB by methods 1 or 2 were included, to avoid circularity. For reports of diagnostic accuracy of proposed biomarkers, only cases diagnosed in the absence of the biomarker of interest were included, to avoid circularity. For inclusion, it was necessary that data were available relating exclusively to MCI-LB participants (i.e., not data that included MCI-LB and DLB or MCI-LB and Parkinson’s disease [PD]-MCI mixed together).

Exclusion criteria: cohorts only reporting Parkinson’s disease PD-MCI (defined by the 1-year rule) ^3^; cross-sectional or longitudinal cohorts of idiopathic RBD without MCI, or mixed RBD with/without MCI; reviews; editorials; case studies or clinical case series with fewer than 10 cases.

### 2.2 | Information sources

PubMed, MEDLINE, and Embase were searched for all English Language publications on 28 September 2022. The search strategies were as follows:

#### PubMed:

((impairment, mild cognitive[MeSH Terms]) OR (impairments, mild cognitive[MeSH Terms]) OR (cognitive impairments, mild[MeSH Terms]) OR (cognitive impairment, mild[MeSH Terms]) OR (mild cognitive impairment[MeSH Terms]) OR (prodromal symptoms[MeSH Terms)) AND ((bodies, lewy[MeSH Terms]) OR (body, lewy[MeSH Terms]) OR (cortical lewy body disease[MeSH Terms]) OR (dementia, lewy body[MeSH Terms]) OR (diffuse lewy body disease[MeSH Terms]) OR (alpha synuclein[MeSH Terms]) OR (synuclein[MeSH Terms]) OR (synucleins [MeSH Terms]))

#### MEDLINE:

(Subject headings: “Lewy bodies” OR “Lewy body disease” OR “Synucleins” OR “alpha-synuclein”) AND (Key words: “MCI” OR “mild cognitive impairment” OR Subject Heading: “Prodromal Symptoms”)

#### Embase:

(Subject headings: “Lewy body” OR “diffuse Lewy body disease” OR “alpha synuclein” OR “Synuclein”) AND (Subject heading: “mild cognitive impairment” OR “prodromal symptom”)

### 2.3 | Selection process

A team of four reviewers evaluated articles to ensure rapid assessment (PCD, CC, DF, AP). At the title and abstract screening stages, each article was independently assessed by two reviewers. In cases of disagreement, a third reviewer reviewed the article for inclusion or exclusion. At the full-text screening stage, all articles were independently reviewed by two reviewers. In cases of disagreement, the two reviewers met to discuss the article. Where a decision on inclusion/exclusion could not be reached, a third reviewer made the final decision.

### 2.4 | Data collection process, data items, and effect measures

Data were extracted by a single reviewer for each article. Data were extracted for the percentage prevalence of diagnostic features in MCI-LB and any comparison MCI groups. Data were also extracted for the predictive validity of diagnostic features (i.e., whether the feature was associated with later development of DLB). The methodological quality of studies was assessed with the CASP checklist for case control studies,^5^ by a single researcher (AH), who consulted with a second researcher (DF) in case of doubt.

### 2.5 | Meta-analysis

Data for the meta-analysis was extracted by a single researcher (A.H.). Only features which were reported in three or more independent studies using comparable assessment methods were included in the meta-analysis. Publications from the same research group were closely examined for overlapping data sets. In case of overlap, only data from the largest cohort reporting the frequency of a feature was included in the meta-analysis. Clarification was sought from the authors where required. Articles presumed or known to contain overlapping data are reported together in the Tables.

Variables included in the meta-analysis were size of MCI groups and frequency of features in each MCI group. Using random-effects models, weighted pooled proportions (*p*P) were calculated for each MCI group, and post hoc paired comparisons were performed between the MCI-LB group and other MCI groups with a *p* value <0.05 deemed significant. Heterogeneity in these analyses was assessed through visual inspection of forest plots (Supplementary Figures) and by computing the *I*^*2*^ parameter. *p* values, 95% confidence intervals (CIs), and complementary meta-analytical parameters are provided in Table 2. Analyses were conducted in R version 4.1.2, using the “Meta” package.^6^

## 3 | RESULTS

**FIGURE 1:**
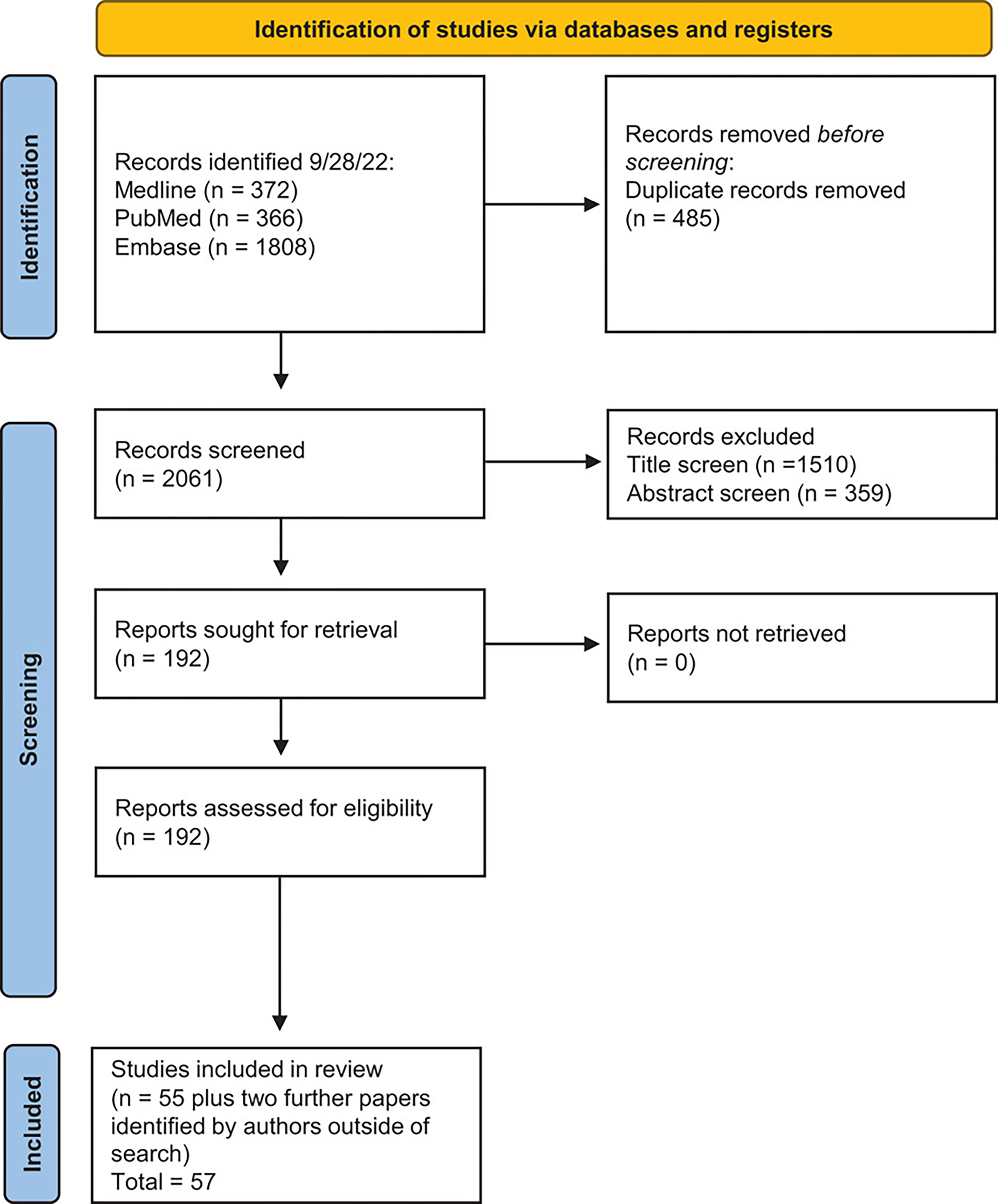
PRISMA flow diagram.

The results of the literature search and screening are displayed in Figure 1. In total, 57 articles were included in the review. The study characteristics and main findings of the articles are displayed in Tables 2–4 and Tables S1 and S2. All the selected studies had adequate methodological quality according to the CASP checklist (Table S3).

### 3.1 | Core clinical features

We identified two studies that reported the prevalence of core clinical features in MCI participants who later had *post mortem* confirmation of LB disease ^7, 8^ (Table S1). Additionally, 12 articles reported rates of core clinical features in cohorts of people with MCI-LB who later met the criteria for a clinical diagnosis of DLB.^9–20^ Our meta-analysis of these studies (Table 2) showed that rates of all four core features were significantly higher in MCI-LB compared with MCI-AD and stable MCI. In MCI-LB, the pooled proportions were highest for parkinsonism (68%) and RBD (60%), whereas cognitive fluctuations (36%) and visual hallucinations (27%) were less common. The pooled proportions of all features were low in MCI-AD (1%–6%) and stable MCI (3%–18%).

The diagnosis of MCI-LB has been demonstrated to be associated with a later diagnosis of DLB in a longitudinal cohort.^21^ Within this cohort including MCI-LB and MCI-AD cases, a higher number of diagnostic features present at the MCI stage was found to predict the development of dementia (hazard ratio 1.3, 95% CI 1.1–1.6), with fluctuations and visual hallucinations showing the strongest relationship.^22^ However, another longitudinal cohort found that, within an MCI-LB cohort, the presence of core clinical features did not predict conversion to dementia (hazard ratio 1.1, 95% CI 0.8–1.5) ^23^ (Table S1).

### 3.2 | Supportive clinical features

Neuropsychiatric symptoms: rates of neuropsychiatric supportive symptoms in the MCI-LB criteria (hallucinations in non-visual modalities, systematized delusions, apathy, anxiety, and depression) were reported in up to seven studies.^23–29^ When comparing MCI-LB with MCI-AD, our meta-analyses showed that the pooled prevalence of anxiety (31% vs. 18%), depression (37% vs. 22%), apathy (47% vs. 23%) and delusions (11% vs. 4%) was higher in MCI-LB (Table 2). We found no difference for non-visual hallucinations (7% vs. 3%), though this feature was only recorded in three studies (Table 2).

Autonomic symptoms: Three studies have systematically surveyed autonomic symptoms in MCI-LB ^25, 30, 31^ (Table S2). The symptoms enquired about varied between the three studies. All three found higher rates of autonomic features in MCI-LB than MCI-AD, including gastrointestinal, genitourinary, secretomotor, and cardiovascular symptoms. Our meta-analysis showed that the pooled proportions of constipation (56% vs. 25%) and difficulty emptying bladder (37% vs. 14%) were higher in MCI-LB compared with MCI-AD (Table 2). Two studies examined the discriminant ability of overall scores in autonomic symptom scales to differentiate between MCI-LB and MCI-AD, finding a similar area under the receiver operating characteristic (AUROC) curve of 0.68 using the Composite Autonomic Symptoms score ^30^ and 0.76 using the Scales for Outcomes in Parkinson’s Disease - Autonomic Dysfunction.^31^

Autonomic signs: Rates of orthostatic hypotension varied considerably between two studies in MCI-LB (19%, 43%) and MCI-AD (18%, 26%).^23, 30^ Other studies have found lower heart rate variability ^19^ and abnormal blood pressure responses to the Valsalva maneuver, but not abnormal heart rate responses in MCI-LB compared with MCI-AD.^32^

Hyposmia: In one study, self-reported loss of smell was common in MCI-LB (44%) and MCI-AD (19%).^33^ Two studies have measured olfactory function using standardized tests.^20, 34^ Both studies found poorer olfactory function in MCI-LB compared with MCI-AD, with an AUROC of 0.67 ^34^ and 0.85 ^20^ (Table S2).

Hypersomnia: Daytime sleepiness was present in the majority of MCI-LB participants in two studies (56, 67%), whereas also being relatively frequent in MCI-AD (29, 38%).^25, 33^

Other supportive features: One study has reported that frequent falls were more common in MCI-LB patients (43%) than MCI-AD (11%), but subjectively reported balance problems, dizziness/fainting and transient loss of consciousness were not.^33^ We did not find any reports of rates of prolonged/recurrent delirium or sensitivity to antipsychotic agents in MCI-LB.

### 3.3 | Proposed biomarkers (Table 3)

Striatal dopaminergic imaging: We identified data reporting the diagnostic accuracy of dopaminergic imaging in a total of 121 possible or probable MCI-LB patients.^35–37^ In one publication that combined data from two cohorts, 40/61 probable MCI-LB had a positive scan (sensitivity 66%; 95% CI 52–77%), compared with 7/26 possible MCI-LB and 5/57 MCI-AD (specificity 88%; 95% CI 76–95%).^37^ 83% of patients with MCI-LB and a positive ^123^I-FP-CIT scan had parkinsonism, compared to 55% in those with a normal ^123^I-FP-CIT scan. In this study, the images were visually rated, with semi-quantitative specific binding ratios (SBRs) used to support visual rating.

Another study investigated semi-quantitative specific binding ratio in an MCI-LB cohort using an SBR z-score threshold of −0.82 for abnormality, determined from an autopsy cohort.^38^ 22/34 (65%) of the MCI-LB cohort were below this threshold.^35^

Cardiac MIBG: Only one study with 37 probable MCI-LB and 43 MCI-AD has reported the accuracy of cardiac denervation as a diagnostic tool in MCI-LB.^39^ The sensitivity to detect probable MCI-LB was 59% (95% CI 42–75%) and the specificity was 88% (75–96%).

Combining data from FP-CIT and MIBG imaging in the same cohort, requiring both scans to be abnormal resulted in a sensitivity to detect MCI-LB of 50%, with a specificity of 100%.^39^

Polysomnography: No studies were found that directly reported the rates of REM sleep without atonia in MCI-LB. A longitudinal study of people with polysomnography confirmed RBD identified an MCI stage in 25/29 people who later developed DLB.^40^ The median duration of the MCI phase was 2 years. A similar longitudinal cohort of people with polysomnography confirmed RBD found that MCI was a risk factor to develop dementia first rather than parkinsonism first in those who developed a neurodegenerative disease.^41^

### 3.4 | Potential biomarkers (Table 4)

Preservation of medial temporal lobe structures: visually rated medial temporal lobe atrophy scores in MCI-LB did not differ with scores in controls or stable MCI in three cohorts ^23, 42–46^ Two of these cohorts also found no difference between MCI-AD and MCI-LB ^23, 42–44, 46^ One cohort found greater atrophy in MCI-AD,^45^ although the degree of difference was modest (mean medial temporal atrophy [MTA] score 1.6 [SD 0.9] in MCI-AD compared with 1.2 [0.7] in MCI-LB).

Hippocampal volume loss was reported in MCI-AD and MCI-LB compared with controls using an automated measure, but no difference between MCI-AD and MCI-LB was found.^47^ When performing voxel-based analyses across the whole brain, the same study reported volume loss in the right hippocampus in MCI-LB and MCI-AD compared with controls, but no difference between MCI-AD and MCI-LB.^47^ In another cohort, there were no differences between MCI-LB and controls or MCI-AD in gray matter volume of medial temporal lobe structures,^48, 49^ whereas an analysis of cortical thinning in the same cohort identified a small area of greater thinning in MCI-AD compared to MCI-LB in the left parahippocampal region.^46^

In a longitudinal study, 85% of MCI cases that progressed to DLB had normal hippocampal volumes, whereas 61% of MCI cases that progressed to AD dementia had abnormal volumes.^16^ Similarly, in a longitudinal study comparing brain atrophy subtypes in converters from MCI to dementia, converters to DLB were over-represented in two subtypes with no hippocampal atrophy (the hippocampal sparing and minimal/no atrophy subtypes), with 60% of DLB cases categorized to these subtypes, compared with 29% of converters to AD.^50^

Insular thinning and gray matter volume loss: Cortical thickness and gray mater volumes have been compared using voxel-wise analysis in MCI-LB.^46–48^ Cortical thinning was found in an area of the right insula in MCI-LB compared with MCI-AD.^46^ In the same cohort, gray matter volume loss was also identified in the insula bilaterally in MCI-LB compared with controls, but not compared with MCI-AD.^48, 49^ Another cohort found insula volume was decreased in both MCI-LB and MCI-AD compared with controls, but found no significant difference between MCI-AD and MCI-LB in a region of interest analysis and no difference between the groups in the insula using a voxel-wise analysis.^47^

Low occipital uptake on metabolism/perfusion imaging: MCI-LB patients have consistently demonstrated reduced fluorodeoxyglucose (FDG) PET uptake in posterior brain areas ^15, 17, 51–54^ One recent study found that visual rating of FDG PET demonstrated an accuracy of 77% to differentiate MCI-LB from MCI-AD,^52^ whereas another found that 50% of MCI-LB had primary visual cortex hypometabolism, compared with 24% of MCI-AD.^17^ Another study found that using the cingulate:cuneus/precuneus ratio demonstrated sensitivity of 59% and specificity of 90% to differentiate MCI-LB from MCI-AD.^15^

A similar pattern of regional brain changes has been reported in brain perfusion studies.^55–57^ The posterior cingulate:precuneus ratio measured by MRI arterial spin labeling (similar to the cingulate:cuneus/precuneus ratio above) was significantly greater in MCI-LB compared with controls, but not when compared with MCI-AD.^55^

Quantitative electroencephalography (EEG) slowing and dominant frequency variability: several studies have demonstrated evidence of quantitative resting-state EEG slowing in MCI-LB relative to controls and/or MCI-AD, including lower dominant/peak frequency,^58–60^ increased theta power,^10, 58^ increased delta power,^60^ and decreased alpha and beta power.^58, 59^ Similar changes were reported in MCI with polysomnography confirmed RBD (probable MCI-LB) compared with controls and MCI without RBD.^61^ Greater dominant frequency variability has also been observed in MCI-LB compared with controls/MCI-AD in one study,^10^ with another study finding no statistically significant difference.^59^

When discriminating MCI-LB from MCI-AD, a range of EEG measures were effective in one study, including theta power (AUROC 0.94, 95% CI 0.88–0.99), beta power (0.91, 0.84–0.98), and theta:alpha ratio (0.92, 0.85–0.99).^58^ EEG changes also predicted conversion to dementia in this study. Another study found AUROC to differentiate MCI-LB and MCI-AD ranging from 0.60 to 0.71 for the same measures,^59^ though theta:alpha power ratio was associated with an increased risk of transition to dementia in the same cohort.^62^ A third study found two features that discriminated between MCI-LB and MCI-AD with an AUROC greater than 0.7: parietal delta power (AUROC 0.72) and temporal delta power (AUROC 0.71).^60^ A longitudinal study categorized EEGs based on abnormalities associated with DLB (reduced dominant frequency <8Hz, increased dominant frequency variability >1.5 Hz). These abnormalities were present in 100% of MCI-LB participants at baseline, whereas these changes were present in only 7% of MCI-AD.^10^ These findings remained stable over the course of the study.

## 4 | DISCUSSION

This article presents a systematic review and meta-analysis of the evidence for the 2020 diagnostic criteria for MCI-LB.^3^ There has been a substantial increase in the evidence for the clinical and biomarker presentation on MCI-LB since the diagnostic criteria were submitted for publication in October 2019. Half of the articles cited in this review were published since this time. Therefore, this is an appropriate time to review the extent to which the new evidence supports the published criteria.

### 4.1 | Core clinical features

Core clinical features are used to diagnose MCI-LB and DLB. When considering the prevalence of core clinical features in MCI-LB, we included articles with *post mortem* confirmation of LB pathological changes or longitudinal follow-up with a confirmed clinical diagnosis of DLB, while we excluded articles with a cross-sectional clinical diagnosis of MCI-LB, to avoid circularity. There were only two studies with *post mortem* confirmation (*n* = 17 MCI-LB) and only one of these studies reported rates of RBD and hallucinations. It is difficult to draw any conclusions from this limited data. However, from previous *post mortem* studies in DLB, we know that all four core clinical features are predictive of the presence of LB pathology.^63^

Longitudinal clinical cohorts found highly variable rates of core features, but all four core features were more common in MCI-LB than MCI-AD and stable MCI (Table 2). The symptoms were highly specific to MCI-LB when compared to MCI-AD. However, some cohorts reported relatively high rates of parkinsonism in stable MCI (pooled prevalence 18%), though this was still substantially lower than those in MCI-LB (pooled prevalence 68%).

Parkinsonism and RBD were the most common symptoms in MCI-LB, with visual hallucinations and fluctuations being less common. The rates of these symptoms (pooled prevalence 27%–68%) were lower than the rates observed in DLB, where rates ≥ 75% are observed over the course of the disease.^63^ As a result, the sensitivity of the clinical core features in the MCI-LB diagnostic criteria is likely to be lower than in the DLB criteria.

The lower rates of visual hallucinations may reflect that this symptom tends to develop later than the other three core features of DLB.^64^ Lower rates of cognitive fluctuations may reflect later onset of this symptom, difficulty in assessing fluctuations in early disease stages, or a misattribution of symptoms to a non-neurodegenerative cause (e.g., recurrent delirium).

The heterogeneity index I^2^ in these analyses was relatively low but we still observed variability across studies in reported rates of core features. This variability was expected and may be due to differences in participant recruitment (e.g., from movement disorders services or memory services) and differences in the assessment of core features (e.g., clinician judgement or thresholds in clinical scales). Studies relying on a clinical diagnosis of DLB at the dementia stage, rather than *post mortem* confirmation, will inevitably exclude people with DLB who do not present with typical clinical symptoms. As such, they may overestimate the rates of clinical features in the MCI stage. Larger cohorts of *post mortem* cases with clinical characterization during MCI will be needed to confirm the validity of the core clinical features for MCI-LB.

### 4.2 | Supportive clinical features

The supportive clinical features are too numerous to deal with individually, but some general conclusions can be made. First, in this systematic review and meta-analysis we observe that none of the supportive clinical features has demonstrated the sensitivity or specificity across different studies to suggest that it should be promoted to the level of a core clinical feature for the diagnosis of MCI-LB. Second, despite their limited diagnostic accuracy, supportive clinical features are very common in MCI-LB, and should be enquired about in clinical encounters to support diagnosis and identify potentially treatable symptoms to improve the quality of life of the person with MCI-LB.

From our meta-analysis, there is clear evidence for the inclusion of anxiety, apathy, depression, delusions, constipation, and difficulty emptying the bladder as supportive symptoms of MCI-LB. There is also consistent evidence of increased overall autonomic symptoms in MCI-LB compared with MCI-AD.^25, 30, 31^

Non-visual hallucinations and delusions are not commonly reported in MCI-LB. The low rates of symptoms of psychosis in MCI-LB may be because these features tend to present later in disease, or because MCI-LB cohorts are currently not identifying cases with a concurrent psychiatric presentation.^65^ Furthermore, the presence of psychiatric diagnoses such as major depressive disorder and primary psychotic disorders are often exclusion criteria in MCI cohorts, therefore there is a risk that the prevalence of these symptoms is systematically and artificially reduced in these cohorts.

Future research should focus on whether combinations of symptoms or the use of clinical scales to measure severity of symptoms, such as constipation and hyposmia, can improve the identification of MCI-LB. These could form important screening questions during clinical assessment or for participant recruitment for MCI-LB studies and clinical trials.

### 4.3 | Proposed biomarkers

Our systematic review revealed limited evidence for the diagnostic effectiveness of dopaminergic imaging and cardiac MIBG in MCI-LB. The studies available suggest that specificity is high in probable MCI-LB compared with MC-AD (88%), but sensitivity is lower (59%–66%).^35, 37, 39^ Unfortunately, the sensitivity in possible MCI-LB appears to be lower than probable MCI-LB.^37, 39^ Further longitudinal studies are needed to understand the predictive validity of these biomarkers, particularly in possible MCI-LB. The potential for relatively high rates of false negative scans in MCI-LB may limit the utility of these biomarkers in research and clinical practice.

From longitudinal studies, it is clear that people with clinical RBD and polysomnography confirmed REM sleep without atonia, along with MCI (by definition probable MCI-LB under the current criteria) are at very high risk of conversion to DLB.^40, 41^ Furthermore, the presence of RBD has been associated with faster cognitive decline in LB diseases.^66^

### 4.4 | Potential biomarkers

Relative preservation of medial temporal lobe structures on structural imaging: Cross-sectional comparisons of medial temporal lobe atrophy in MCI-LB and MCI-AD have reported no significant differences between the groups in most studies. This suggests that visual rating and volumetric measures of medial temporal lobe atrophy might not effectively discriminate between MCI-LB and MCI-AD. Despite this, the absence of hippocampal atrophy in MCI has been reported to be associated with progression to DLB.^16, 50^ However, preserved hippocampal volume was also common in MCI-AD (39%) and stable MCI (76%).^16^ This would need to be considered before using preserved hippocampal volume as a method to enrich MCI samples with MCI-LB cases. The new signature of preserved hippocampal volume in combination with presence of cortical atrophy ^67^ may be more specific but it has only been investigated in one MCI-LB study so far.^50^

Insular thinning and gray matter volume loss on MRI: There have been inconsistent reports of gray matter changes in the insula in MCI-LB compared with MCI-AD and controls. The diagnostic accuracy of this biomarker remains to be determined.

Beyond the findings for medial temporal lobe and insular cortex, visual assessment of global cortical atrophy and posterior atrophy were associated with a shorter time of progression to DLB in MCI-LB.^23^ This may be useful to enrich cohorts with dementia converters, though this finding requires replication.

At the group level, MCI-LB demonstrates posterior cerebral hypometabolism and hypoperfusion when compared with MCI-AD and controls ^15, 17, 51–57^ Visual rating and quantitative measures aiming to measure the cingulate island sign using FDG PET have reported accuracy values similar to that found for the proposed biomarkers.^15, 17, 52^ However, a standardized method of reporting or analyzing these scans has not yet been established. This should be an area of focus for future research. Other quantitative measurements have also been reported, and deserve further investigation, including medial temporal:substantia nigra and occipital:medial temporal ratios.^15, 55^

Quantitative EEG showing slowing and dominant frequency variability: resting state EEG studies have demonstrated consistent evidence of slowing (i.e., decrease in frequency of the dominant power, higher power at delta-theta bands and lower power at alpha-beta bands) in MCI-LB compared with controls and MCI-AD. The discriminant ability of EEG is less clear, with some studies demonstrating excellent discriminant ability ^10, 58^ but not others.^59, 60^ EEG abnormalities may increase the risk of conversion to dementia.^58, 62^ An EEG marker that predicts conversion to dementia could be useful in enriching clinical trial cohorts, particularly in studies where conversion to dementia is an outcome measure. At present, the evidence available supports the inclusion of EEG slowing as a potential biomarker. In contrast, there is less consistent evidence for dominant frequency variability as a biomarker for MCI-LB.^10, 59^

Interestingly, EEG slowing was associated with visual hallucinations and poorer cognitive performance.^59^ This raises the possibility that EEG may be a more effective biomarker in MCI-LB with more significant cognitive impairment and early visual hallucinations (“top down” disease spread), whereas FP-CIT and cardiac MIBG may be most effective in those with early RBD and parkinsonism (“bottom up” disease spread). This should be tested in future research.

### 4.5 | Strengths and limitations

This is the first systematic review and meta-analysis of the evidence for the research diagnostic criteria for MCI-LB. Our report includes a significant amount of new information that was unpublished at the time the criteria were written. We hope that the findings will be useful to researchers who are developing MCI-LB cohorts.

The limitations of this systematic review include the variability between studies in case ascertainment and the method of determining the presence of diagnostic features. LB disease biomarkers were often used in diagnosis, but most cohorts did not use biomarkers to confirm Alzheimer’s disease pathology in the MCI-AD groups. The clinical diagnosis on MCI-AD has low diagnostic specificity.^68^ With longer-term clinical follow-up of these cohorts and, crucially, *post mortem* confirmation of diagnosis, greater differences between MCI-LB and MCI-AD may become evident.

We found little evidence for one of the most critical aspects of the diagnostic criteria – proposed biomarkers. This was in part due to our selection criteria, which excluded articles that included the symptom or biomarker of interest as part of the diagnostic process. For example, if abnormal dopaminergic imaging was used in the diagnosis MCI-LB, we chose not to use this cohort to identify the prevalence of abnormal dopaminergic imaging in MCI-LB. This was crucial, to avoid circularity in our evidence, and increases the reliability of the findings presented.

While all included studies were of an overall good quality, the CASP criteria indicated that matching of demographic variables between groups and/or correction for these confounders should be considered in future studies. In addition, investigation of the potential impact of medications on clinical features and disease biomarkers should also be considered.

### 4.6 | Future directions

The diagnosis of prodromal disease is challenging, as by its nature, it reflects a stage of disease where symptoms and biomarker results will be less clear. As a result of recent advances in imaging and biofluid analysis, diagnosis in Alzheimer’s disease is moving toward a biological definition.^69^ Assays have been developed to detect abnormal alpha-synuclein in skin, cerebrospinal fluid (CSF), and blood,^70–72^ although the development of alpha-synuclein imaging ligands is less advanced.^73^ A widely available, sensitive and specific test for synucleinopathy would revolutionize the in-vivo diagnosis of LB disease at all clinical stages and would represent a significant advance for the field. Assays to detect abnormal alpha-synuclein in the skin and CSF are already commercially available in some regions.^74^ There is evidence for the accuracy of CSF seeding assays in differentiating MCI-LB from controls and MCI-AD.^70^ We are unaware of evidence for the detection of alpha-synuclein in skin in MCI-LB, but there is evidence that skin alpha-synuclein can differentiate idiopathic RBD from controls.^75^

The identification of MCI-LB may benefit from a two-stage process, where screening measures of clinical symptoms and signs are used to detect those at risk, followed by more specialized biomarkers for diagnostic confirmation. There are many potential avenues to investigate for initial screening measures, including simple screening questionnaires that may include core and suggestive clinical features reviewed here. Diagnostic tools have been validated for the dementia stage of DLB, but there are none, at present, for MCI-LB.^76^ The aim of these tests would be to achieve high sensitivity, with acceptable specificity. To date, most clinical features achieve acceptable specificity with suboptimal sensitivity, therefore a combination of factors (e.g., combining neuropsychiatric, autonomic and motor features) may improve this. Artificial intelligence tools may help to increase the accuracy of single biomarkers and facilitate combining multiple clinical and biomarker features, to improve diagnostic accuracy.^77^ Novel digital biomarkers (e.g., actigraphy, computerized cognitive testing) could be developed to identify diagnostic features at an earlier stage, such as cognitive fluctuation, motor changes, and REM sleep behavior disorder.

More advanced MRI modalities, such as fMRI connectivity ^78^ and quantitative susceptibility mapping,^79^ do not appear to currently be specific markers for MCI-LB, but interest in their potential to assess neurodegeneration in DLB has recently been rekindled.^80^ Other modalities remain to be investigated, such as susceptibility-weighted and neuromelanin imaging of the substantia nigra ^81^ and voxel-wise whole-brain dopaminergic imaging.^82^ Novel EEG measures are also currently under investigation. In spatiotemporal analysis, MCI-LB show more frequent alterations in microstates than controls.^83^ However, alpha reactivity on eye opening ^84^ and on measures of inter- and intra-hemispheric alpha connectivity ^85^ do not appear to differ between MCI-LB and MCI-AD at present.

### 4.7 | Recommendations

The DLB research community and funders should support ongoing MCI-LB cohorts and the development of new MCI-LB cohorts in order to increase the evidence available on which to base our diagnostic criteria for this disease stage and validate the criteria for routine clinical use. This review has focused on MCI-LB, but research is also needed into presentation prior to the onset of MCI, and other prodromal presentations, such as psychiatric-onset and delirium-onset DLB, which may occur with or without concurrent MCI.^3, 86, 87^ Recruitment strategies and inclusion/exclusion criteria for longitudinal cohorts should seek to include the broad spectrum of presentations of prodromal DLB.

MCI-LB cohorts should, wherever possible, collect dopaminergic imaging and cardiac MIBG in order to determine the accuracy of these biomarkers and their predictive validity at this disease stage. Established cohorts which have not yet published articles that allow extraction of the sensitivity and specificity of these biomarkers when they are excluded from the diagnostic algorithm (i.e., avoiding circularity) should consider doing so. Similarly, cohorts with data on core and suggestive clinical features should present these data in future publications.

Promising results have been reported for quantitative EEG and FDG PET. Further studies are needed to determine their diagnostic utility.

Wherever possible, MCI cohorts should include the option of brain donation for participants. This will allow the comparison of diagnostic features and biomarkers with pathological diagnosis.

Cohorts should seek to co-ordinate assessment schedules and consider establishing questionnaires to ascertain the presence or absence of clinical features in order to improve comparability between centers. Where possible, this should include the collection of data on olfactory function and autonomic dysfunction. Ideally, large cohorts investigating subjective cognitive impairment and MCI should collect data on LB disease features, given that a significant proportion of their cases are likely to have LB disease.^88^

Co-pathology is common in the dementia *post mortem* studies, and there is evidence that the presence of co-pathology can influence clinical presentation.^89^ The influence of AD and other co-pathologies on clinical presentation and disease progression in MCI-LB should be investigated.

The accuracy of the MCI-LB diagnostic criteria to differentiate between MCI-LB and atypical AD presentations and neurodegenerative diseases other than AD that may present with MCI (e.g., vascular dementia, frontotemporal dementia, progressive supranuclear palsy, and corticobasal degeneration) should be investigated.

Many of the cohorts included in this review were predominantly male and of European descent. The difference in clinical presentation and disease biomarkers in males and females and in diverse populations with MCI-LB should be investigated.^90^

## 5 | CONCLUSIONS

The available evidence largely supports the current diagnostic criteria for MCI-LB, although there is a limited amount of evidence for proposed biomarkers, at present. The research criteria for MCI-LB present a rational framework that will allow sites across the world to recruit longitudinal cohorts of MCI-LB. Over the coming years, further evidence should emerge to help refine the diagnostic criteria to improve their sensitivity to identify MCI-LB and understand how best to apply them in clinical practice and research.

## Supplementary Material

Appendix 1

Supplementary Figures

Supplementary Tables

Supplementary Information

## Figures and Tables

**TABLE 1.** Diagnostic features from the diagnostic criteria for mild cognitive impairment with Lewy bodies (McKeith et al. 2020)^3^

**Core clinical features**
• Fluctuating cognition with variations in attention and alertness.
• Recurrent visual hallucinations.
• REM sleep behavior disorder.
• One or more spontaneous cardinal features of parkinsonism.
**Proposed biomarkers**
• Reduced dopamine transporter uptake in basal ganglia demonstrated by SPECT or PET.
• Polysomnographic confirmation of REM sleep without atonia.
• Reduced meta-iodobenzylguanidine (MIBG) uptake on myocardial scintigraphy.
**Supportive clinical features**
Severe sensitivity to antipsychotic agents; postural instability; repeated falls; syncope or other transient episodes of unresponsiveness; prolonged or recurrent delirium; autonomic dysfunction; hypersomnia; hyposmia; hallucinations in other modalities; systematized delusions; apathy, anxiety, and depression.
**Potential biomarkers of MCI-LB**
• Quantitative EEG showing slowing and dominant frequency variability.
• Relative preservation of medial temporal lobe structures on structural imaging.
• Insular thinning and gray matter volume loss on MRI.
• Low occipital uptake on perfusion/metabolism scan.
A diagnosis of probable MCI-LB requires the presence of two core clinical features, or one core clinical feature and one or more proposed biomarkers.
A diagnosis of possible MCI-LB requires the presence of one core clinical feature and no proposed biomarkers, or one or more proposed biomarkers with no core clinical features.

**TABLE 2:** Meta-analysis of core and supportive clinical features.

Feature		MCI-LB	MCI-AD	Stable MCI	p MCI-LB vs. MCI-AD	p MCI-LB vs. stable MCI
*Core clinical features*						
Visual hallucinations	Pooled proportion (95% CI)	27% (16–43)	1% (0–5)	3% (2–6)	<0.0001	0.0003
	No. of studies (participants)	9 (153)	6 (258)	4 (309)	-	-
	I^2^ (95% CI)	31% (0–68)	0% (0–75)	0% (0–85)	-	-
Cognitive fluctuations	Pooled proportion (95% CI)	36% (24–50)	3% (1–9)	-	<0.0001	-
	No. of studies (participants)	8 (159)	5 (336)	-	-	-
	I^2^ (95% CI)	56% (4–80)	0% (0–79)	-	-	
Parkinsonism	Pooled proportion (95% CI)	68% (55–79)	6% (2–15)	18% (14–23)	<0.0001	0.02
	CNo. of studies (participants)	9 (142)	6 (256)	3 (301)	-	
	I^2^ (95% CI)	48% (0–76)	0% (0–75)	0% (0–90)	-	
REM-sleep behavior disorder	Pooled proportion (95% CI)	60% (41–76)	4% (1–10)	8% (4–18)	<0.0001	<0.0001
	No. of studies (participants)	8 (167)	6 (359)	5 (391)	-	-
	I^2^ (95% CI)	70% (38–86)	11% (0–77)	70.0% (24–88)	-	-
*Supportive clinical features*						
Anxiety	Pooled proportion (95% CI)	31% (22–42)	18% (13–23)	-	<0.0001	-
	No. of studies (participants)	7 (331)	6 (1754)	-	-	-
	I^2^ (95% CI)	69% (32–86)	26% (0–69)	-	-	-
Depression	Pooled proportion (95% CI)	37% (25–50)	22% (20–24)	-	<0.0001	-
	No. of studies (participants)	7 (331)	6 (1754)	-	-	-
	I^2^ (95% CI)	75% (46–88)	28% (0–70)	-	-	-
Apathy	Pooled proportion (95% CI)	47% (32–62)	23% (16–33)	-	<0.0001	-
	No. of studies (participants)	7 (331)	6 (1754)	-	-	-
	I^2^ (95% CI)	86% (72–93)	90% (81–95)	-	-	-
Delusions	Pooled proportion (95% CI)	11% (8–15)	4% (3–5)	-	0.002	-
	No. of studies (participants)	6 (309)	5 (1675)	-	-	-
	I^2^ (95% CI)	0% (0–75)	0% (0–79)	-	-	-
Non-visual hallucinations	Pooled proportion (95% CI)	7% (3–17)	3% (1–9)	-	0.42	-
	No. of studies (participants)	3 (119)	3 (101)	-	-	-
	I^2^ (95% CI)	46% (0–84)	0% (0–90)	-	-	-
Constipation	Pooled proportion (95% CI)	57% (45–65)	25% (20–32)	-	0.006	-
	No. of studies (participants)	3 (90)	3 (205)	-	-	-
	I^2^ (95% CI)	58% (0–88)	0% (0–90)	-	-	-
Difficulty emptying bladder	Pooled proportion (95% CI)	37% (21–56)	14% (7–26)	-	0.0001	-
	No. of studies (participants)	3 (90)	3 (205)	-	-	-
	I^2^ (95% CI)	76% (21–93)	77% (24–93)	-	-	

Abbreviations: CI, confidence interval; MCI-LB, mild cognitive impairment with Lewy bodies; MCI-AD, mild cognitive impairment due to Alzheimer’s disease; REM, rapid eye movement.

**TABLE 3:** Proposed biomarkers.

Author, year Method of diagnostic classification	Study sample	Probable MCI-LB (% abnormal)	Possible MCI-LB (% abnormal)	MCI-AD (% abnormal)
*Dopaminergic imaging*				
Chen 2021 ^35^ Clinical (MCI-LB)	FP-CIT SPECT 34 poss or prob MCI-LB	22/34 (65%) MCI-LB showed abnormal imaging (defined by semi-quantitative putamen z-score < −0.82). No comparison with other diagnostic groups		
Roberts 2021 ^37^ (Thomas 2018) ^36^ Clinical (MCI-LB/DLB)	FP-CIT SPECT 61 Prob MCI-LB 26 Poss MCI-LB 57 MCI-AD	66	27	12
*Cardiac MIBG*				
Roberts 2021 ^39^ Clinical (MCI-LB/DLB)	37 Prob MCI-LB 15 Poss MCI-LB 43 MCI-AD	59	13	12
*PSG-RBD*				
Iranzo 2014 ^40^ Clinical (DLB)	178 RBD 29 converted to DLB	In 25/29 of RBD converted to DLB a MCI phase was recognized (with a median of 2 years from MCI to dementia with no mention of other core and supportive features).		
Postuma 2019 ^41^ Clinical (DLB)	1280 RBD patients 146 converted to dementia	iRBD cases that developed disease were more likely to have MCI than those who did not (55% vs. 16%). In those that developed disease, MCI was associated with higher risk of dementia (84% of ‘dementia-first’ had MCI at baseline vs. 26% in ‘parkinsonism first’)		

*Note*: Articles listed in alphabetical order by first author.

Abbreviations: AD, Alzheimer’s disease; FP-CIT, 2*β*-carbomethoxy-3*β*-(4-iodophenyl)-N-(3-fluoropropyl) nortropane; iRBD, idiopathic rapid eye movement sleep behavior disorder; MCI-LB, mild cognitive impairment with Lewy bodies; MIBG, meta-iodobenzylguanidine; PET, positron emission tomography; Prob, probable; Poss, possible; RBD, rapid eye movement sleep behavior disorder; SPECT, single photon emission computed tomography; Method of diagnostic classification: Clinical (MCI-LB) - clinical diagnosis of MCI-LB; Clinical (DLB) - clinical diagnosis of DLB after conversion to dementia, data reported are from MCI phase.

**TABLE 4:** Potential biomarkers.

Author, year Method of diagnostic classification	Study sample	Key findings
*Structural imaging of medial temporal lobe structures and insula*		
Blanc 2015, 2016 and Roquet 2017 ^46, 48, 49^ Clinical (MCI-LB)	28 Prob MCI-LB 27 MCI-AD 33 control (Roquet 2017: 54 MCI-LB, 16 MCI-AD, 22 control)	Cortical thickness (voxel-wise): thinning in left parahippocampal gyrus in MCI-AD v MCI-LB and right insula in MCI-LB v MCI-AD. No differences between MCI-LB and controls after family-wise error correction. Gray matter volume (voxel-wise): loss in left and right insula in MCI-LB compared with controls, no difference when compared with MCI-AD. No difference in medial temporal areas in MCI-LB compared with MCI-AD or controls.
Bousiges 2020 (Bousiges 2016, 2018)^42–44^ Clinical (MCI-LB)	51 Prob MCI-LB 33 MCI-AD 21 control	Medial temporal atrophy (visual rating): no significant difference between MCI-LB and MCI-AD or controls. Majority of MTA scores were 0 or 1 in MCI-LB and MCI-AD.
Firbank 2021 ^47^ Clinical (MCI-LB/DLB)	38 Prob MCI-LB 36 MCI-AD 31 control	Gray matter volume (Region of interest): reduced hippocampal and insula volumes in both MCI-LB and MCI-AD compared with controls. No difference between MCI-LB and MCI-AD. Gray matter volume (voxel-based analysis): Reduced volume in right hippocampus in MCI-LB v control, no differences found in insula. No differences found between MCI-LB and MCI-AD.
Kantarci 2016 ^16^ Clinical (DLB)	20 Prob MCI-LB 61 MCI-AD 79 stable MCI	Preserved hippocampal volume increased risk of conversion to DLB compared with AD (hazard ratio 5.8, 95% CI 1.9–18.0). 15% of MCI cases that converted to DLB had hippocampal atrophy, compared to 61% of cases that converted to AD.
Planche 2021 ^50^ Clinical (DLB)	15 MCI-LB 142 MCI-AD	Comparison of atrophy subtypes in MCI cases that converted to DLB compared to other dementias. DLB were over-represented in hippocampal-sparing (13%) and minimal/no atrophy (47%) subtypes. In MCI-AD, 10% had a hippocampal-sparing subtype and 19% had a minimal/no atrophy subtype.
Siddiqui 2020 ^45^ Clinical (DLB)	28 Probable MCI-LB 27 MCI-AD 28 stable MCI	Medial temporal lobe atrophy scores, mean (SD): MCI-LB 1.2 (0.7), MCI-AD 1.6 (0.9), stable MCI 1.2 (0.7). Scores in MCI-AD were greater than MCI-LB or stable MCI.
Van de Beek 2020 ^23^ Clinical (MCI-LB/DLB)	58 Probable MCI-LB 111 MCI-AD	Medial temporal lobe atrophy scores, median (IQR): MCI-LB 1 (0–1) v MCI-AD 1 (0–2), no significant difference in scores.
*Perfusion/metabolism imaging*		
Firbank 2021 ^55^ Clinical (MCI-LB/DLB)	Arterial spin labelling 32 Prob MCI-LB 30 MCI-AD 28 controls	In MCI-LB compared with controls: decreased perfusion in precuneus/superior patietal, inferior occipital/temporal and inferior parietal areas. Posterior cingulate:precuneus ratio greater in MCI-LB than controls, difference with MCI-AD did not reach significance.
Kantarci 2021 ^15^ Clinical (DLB)	FDG-PET 17 Prob MCI-LB 41 MCI-AD 100 control	MCI-LB demonstrated parieto-occipital hypometabolism when compared to controls. Cingulate island sign ratio: sensitivity 59%, specificity 90% to differentiate MCI-LB and MCI-AD.
Kondo 2016 ^17^ Clinical (DLB)	FDG PET 12 Prob MCI-LB 21 MCI-AD 58 MCI stable	The MCI-LB group had lower metabolism in parietal and precuneus and primary visual cortex than the AD MCI group, and there were no differences in posterior cigulate. 50% of MCI-LB had primary visual cortex hypometabolism, compared to 24% of MCI-AD.
Massa 2022 (Massa 2019)^51, 52^ Clinical (MCI-LB/DLB)	FDG PET 39 Prob MCI-LB (26 converted to DLB) 40 MCI-AD (33 converted to AD)	Compared with controls, decreased perfusion was found in temporo-limbic areas in MCI-AD and parieto-occipital areas in MCI-LB. Scans were visually rated blind to diagnosis with a diagnostic accuracy of 77%. Region of interest-based semi-quantitative measures increased accuracy to 90%.
Roquet 2016 ^49^ Clinical (MCI-LB)	Arterial spin labelling 46 Prob MCI-LB 13 MCI-AD 21 control	In probable MCI-LB areas of relative hypoperfusion found in middle temporal, anterior insula, inferior frontal, superior parietal and superior orbital areas, with relative hyperperfusion in superior frontal area compared to controls. Relative hypoperfusion in fusiform region compared to MCI-AD.
Vendette 2012 ^56^ Clinical (MCI-LB)	99mcTc-Ethylene Cysteinate Dimer PET 10 Prob MCI-LB (polysomnography confirmed RBD) 20 control.	Compared with controls, MCI-LB demonstrated hypoerfusion in middle frontal, cuneus, superior occipital, parieto-occipital, inferior parietal and superior temporal areas, and hyperperfusion in hippocampus, parrahippocampal and paracentral areas.
Yoo 2021 * ^53^ Clinical (MCI-LB)	FDG-PET 20 prob MCI-LB (RBD with MCI) 24 RBD no MCI 13 Control	RBD with MCI showed higher z-scores than controls in a pre-defined brain metabolic pattern derived from cases of PD with RBD, characterized by posterior hypometabolism and hypermetabolism in cortical and subcortical motor areas. There was no difference between RBD with MCI and controls/iRBD in a separate pattern derived from iRBD cases.
Yoon 2022* ^54^ Clinical (MCI-LB/DLB)	FDG-PET 21 prob MCI-LB (RBD with MCI) 19 RBD no MCI 24 Control	Compared with health controls, RBD with MCI demonstrated significant posterior hypometabolism on FDG PET. There was no significant difference between the groups in the cingulate island sign ratio (posterior cingulate:cuneus/precuneus). 7/24 converted (3 to DLB, 4 to PD). Occipital hypometabolism was associated with higher risk of conversion.
*EEG*		
Babiloni 2018 ^60^ Clinical (MCI-LB)	23 Prob MCI-LB 30 MCI-AD 30 control	MCI-LB had marked slowing in individual alpha frequency peak compared with MCI-AD and controls. MCI-LB also demonstrated greater delta activity in frontal, parietal and temporal areas. Parietal delta activity demonstrated an AUC of 0.89 to differentiate MCI-LB from controls and AUC of 0.72 to differentiate MCI-LB from MCI-AD.
Bonanni 2015 ^10^ Clinical (DLB)	20 Prob MCI-LB 14 MCI-AD 8 MCI stable 50 control	EEGs were characterised on the presence or absence of abnormalities associated with DLB (dominant frequency <8 Hz or dominant frequency variability >1.5 Hz). 100% MCI-LB patients showed EEG abnormalities, 93% of MCI-AD had neither of these abnormalities through the course of the study (i.e., a normal EEG at baseline and follow-up).
Hamilton 2022 ^62^ Clinical (DLB)	39 prob MCI-LB 17 poss. MCI-LB 36 MCI-AD 31 Control	5 MCI-AD converted to AD, 9 MCI-LB converted to DLB. Increased theta:alpha ratio associated with increased risk of transition to dementia when controlling for MCI subtype (HR 1.8, 95% CI 1.0–3.4). Differences were no longer significant after controlling for cholinesterase inhibitor use.
Rodrigues Brazète 2013^61^ Clinical (MCI-LB)	23 Prob MCI-LB (polysomnography confirmed RBD) 19 MCI without RBD 37 controls	MCI-LB had a higher slow-to-fast frequency ratio than MCI non iRBD and controls in the parietal, temporal, and occipital regions. MCI-LB had a lower occipital dominant frequency than controls but not MCI without RBD. MCI-LB had higher relative theta power in the parietal, temporal, and occipital regions and lower relative alpha power in the occipital region compared to MCI no iRBD and controls. Furthermore, MCI-LB had higher relative theta power in the frontal and central areas and lower relative beta power in the central, parietal, and temporal regions compared to controls.
Schumacher 2020 (Schumacher 2022)^59,84^ Clinical (MCI-LB/DLB)	39 Prob MCI-LB 36 MCI-AD 31 controls	MCI-LB had greater pre-alpha power and lower beta power, dominant frequency and occipital dominant frequency than MCI-AD and controls. MCI-LB also had greater theta power and lower alpha power than controls. No difference between the groups in theta/alpha ratio or dominant frequency variability. The greatest discriminant ability was found for beta power (0.71, 95% CI 0.51–0.77) and dominant frequency (0.70, 0.58–0.82).
Van der Zande 2020 ^58^ Clinical (MCI-LB/DLB)	37 Prob/poss MCI-LB 67 MCI-AD	MCI-DLB had greater theta power, greater theta:alpha ratio and lower alpha-2 power, beta power and peak frequency than MCI-AD. Several individual EEG measures showed good performance to discriminate MCI-DLB from MCI-AD including theta power (AUC 0.94, 95% CI 0.88–0.99), beta power (0.91, 0.84–0.98) and theta:alpha ratio (0.92, 0.85–0.99). Qualitative visual EEG score was higher in MCI-DLB v MCI-AD. EEG changes predicted conversion to dementia in MCI-LB.

*Note*: Articles listed in alphabetical order by first author.

Abbreviations: AD, Alzheimer’s disease; AUC, area under the curve; CI, confidence interval; EEG, electroencephalography; FDG, fluorodeoxyglucose; HR, hazard ratio; iRBD, idiopathic rapid eye movement sleep behavior disorder; MCI-LB, mild cognitive impairment with Lewy bodies; PET, positron emission tomography; Prob, probable; Poss possible; RBD REM sleep behavior disorder; SPECT, single photon emission computed tomography.

Method of diagnostic classification: Clinical (MCI-LB) - clinical diagnosis of MCI-LB; Clinical (DLB) - clinical diagnosis of DLB after conversion to dementia, data reported are from MCI phase.

* There may be some overlap in cases in these cohorts.

## Highlights

- A meta-analysis of the diagnostic features of MCI-LB was carried out.
- The four core clinical features were more common in MCI-LB than MCI-AD/stable MCI.
- Neuropsychiatric and autonomic features were also more common in MCI-LB.
- More evidence is needed for the proposed biomarkers.
- FDG-PET and quantitative EEG show promise as diagnostic biomarkers in MCI-LB.

## RESEARCH IN CONTEXT

### 1. Systematic Review:

MEDLINE, PubMed, and Embase were searched on 9/28/22 for relevant articles. Articles were included if they presented original data reporting the rates of diagnostic features in mild cognitive impairment with Lewy bodies (MCI-LB).

### 2. Interpretation:

Our review and meta-analysis demonstrated that core clinical features are specific to MCI-LB, but that rates are lower than those observed in dementia with Lewy bodies. Neuropsychiatric and autonomic supportive clinical features are also common in MCI-LB, but less specific to the disease. More data is needed on the proposed biomarkers but that available supports their inclusion. Of the potential biomarkers, fluorodeoxyglucose-positron emission tomography (FDG-PET) and quantitative electroencephalography (EEG) have demonstrated promising results.

### 3. Future Directions:

Future research should identify how we can screen for, and identify, cases of MCI-LB using current tools. New diagnostic tools are urgently needed. A sensitive and specific biofluid marker for Lewy body disease using seeding assays could revolutionize the diagnosis of MCI-LB.
