# Supplementary material for: Research diagnostic criteria for mild cognitive impairment with Lewy bodies: A systematic review and meta-analysis
Source: Alzheimers Dement. Author manuscript; Available in PMC 2023 Dec 4. (PMC10695683; doi:10.1002/alz.13105)
Supplement: Appendix 1 [file NIHMS1944285-supplement-Appendix_1.docx]

**APPENDIX 1**

Collaborators.

Alzheimer’s Association International Society to Advance Alzheimer’s Research and Treatment Lewy Body Dementias Prodromal Working Group Members (in alphabetical order)

Carla Abdelnour

Stanford University School of Medicine, Department of Neurology and Neurological Sciences, Stanford, CA, USA.

Paulo Henrique Ferreira Bertolucci

Department of Neurology and Neurosurgery, Escola Paulista de Medicina, Federal University of São Paulo (UNIFESP), Sao Paulo, Brazil

Bradley F. Boeve

Mayo Clinic Rochester, Rochester, Minnesota, USA

Tinatin Chabrashvili

State University of New York Upstate Medical University, Syracuse, New York, USA

Emma Cunningham

Centre for Public Health, Queen’s University Belfast, Belfast, UK

Fabrizia D’Antonio

Department of Human Neurosciences, Sapienza University of Rome, Rome, Italy

Shubham Dubey

Department of Neurology, Meenakshi Medical College Hospital and Research Institute, Kanchipuram, India

Jakub Hort

Charles University, 2nd Faculty of Medicine, Motol University Hospital, Prague, Czech Republic; International Clinical Research Center, St. Anne’s University Hospital Brno, Brno, Czech Republic; Memory Clinic, Department of Neurology, Charles University, 2nd Faculty of Medicine and Motol University Hospital, Prague, Czech Republic

Ahmad Khundakar

School of Health & Life Sciences, Teesside University, Middlesbrough, UK; National Horizons Centre, Teesside University, Darlington, UK; Translational and Clinical Research Institute, Newcastle University, Newcastle Upon Tyne, UK

Afina W Lemstra

Alzheimer Center Amsterdam, Department of Neurology, Amsterdam Neuroscience, Vrije Universiteit Amsterdam, Amsterdam UMC, Amsterdam, The Netherlands

Iracema Leroi

Global Brain Health Institute, California, USA & Dublin, Ireland; Trinity College Dublin, Dublin, Ireland

James B. Leverenz

Lou Ruvo Center for Brain Health, Neurological Institute, Cleveland Clinic, Cleveland, Ohio, USA

Irene Litvan

Department of Neuroscience, University of California, San Diego, San Diego, California, USA

Ian McKeith

Translational and Clinical Research Institute, Newcastle University, Newcastle upon Tyne, UK

John O’Brien

Department of Psychiatry, University of Cambridge, Cambridge, United Kingdom

Fabricio Oliveira

Elysian Clinic, São Paulo, SP, Brazil

Alessandro Padovani

Department of Clinical and Experimental Sciences, Neurology Unit, University of Brescia

Kathleen Poston

Department of Neurology and Neurological Sciences, Stanford University, Stanford, CA, United States

Federico Rodriguez-Porcel

Department of Neurology, Medical University of South Carolina, Charleston, South Carolina, USA

Arvid Rongve

Department of Research and Innovation, Helse Fonna, Haugesund, Norway; Institute of Clinical Medicine (K1), The University of Bergen, Norway

David Salmon

Department of Neurosciences, University of California, San Diego

Sonja Scholz

Neurodegenerative Diseases Research Unit, National Institute of Neurological Disorders and Stroke, Bethesda, Maryland, USA; Department of Neurology, Johns Hopkins University School of Medicine, Baltimore, Maryland, USA

Aoife Sweeney

Centre for Public Health, Queen’s University Belfast, Belfast, UK

John-Paul Taylor

Translational and Clinical Research Institute, Newcastle University, Newcastle upon Tyne, UK

Jon B. Toledo

Stanley Appel Neurology Department, Houston Methodist Hospital, Houston, Texas, USA

Prabitha Urwyler

Gerontechnology and Rehabilitation, ARTORG Center for Biomedical Engineering, University of Bern, Bern, Switzerland; University Neurorehabilitation Unit, Department of Neurology, Inselspital, Bern University Hospital, Bern, Switzerland

Huali Wang

Dementia Care & Research Center, Peking University Institute of Mental Health (Sixth Hospital), Beijing, China; Beijing Municipal Key Laboratory for Translational Research on Diagnosis and Treatment of Dementia, Beijing, China; Key Laboratory for Mental Health, Ministry of Health (Peking University), Beijing, China

Kathryn Wyman-Chick

HealthPartners Center for Memory and Aging and Struthers Parkinson’s Center, Saint Paul, Minnesota, USA
