# Supplementary material for: Research diagnostic criteria for mild cognitive impairment with Lewy bodies: A systematic review and meta-analysis
Source: Alzheimers Dement. Author manuscript; Available in PMC 2023 Dec 4. (PMC10695683; doi:10.1002/alz.13105)

**Core clinical features**

**Visual hallucinations**

**Supplementary Figure 1A. Visual hallucinations: MCI-LB**
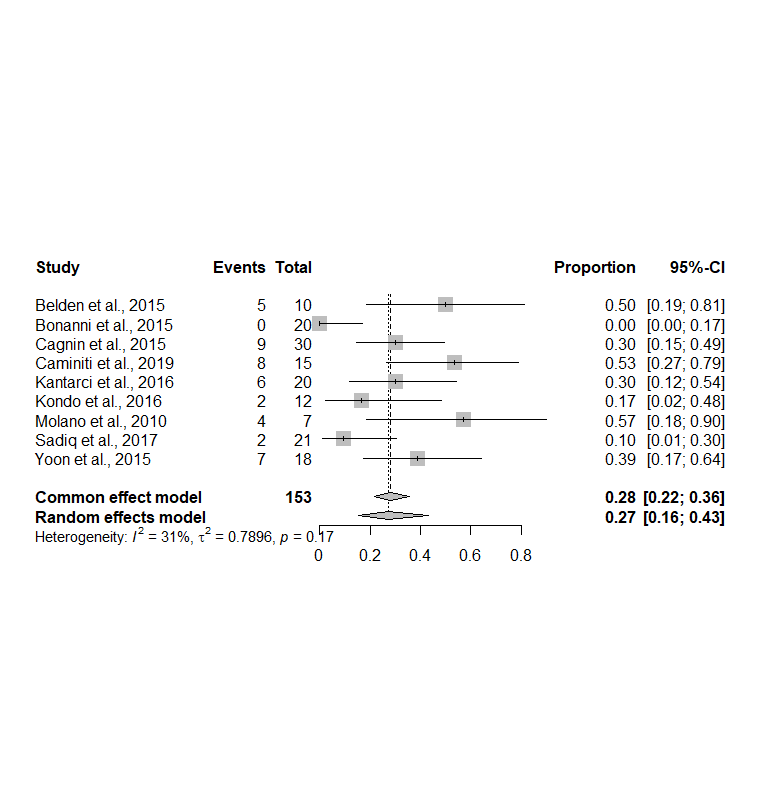


**Supplementary Figure 1B. Visual hallucinations: MCI-AD**
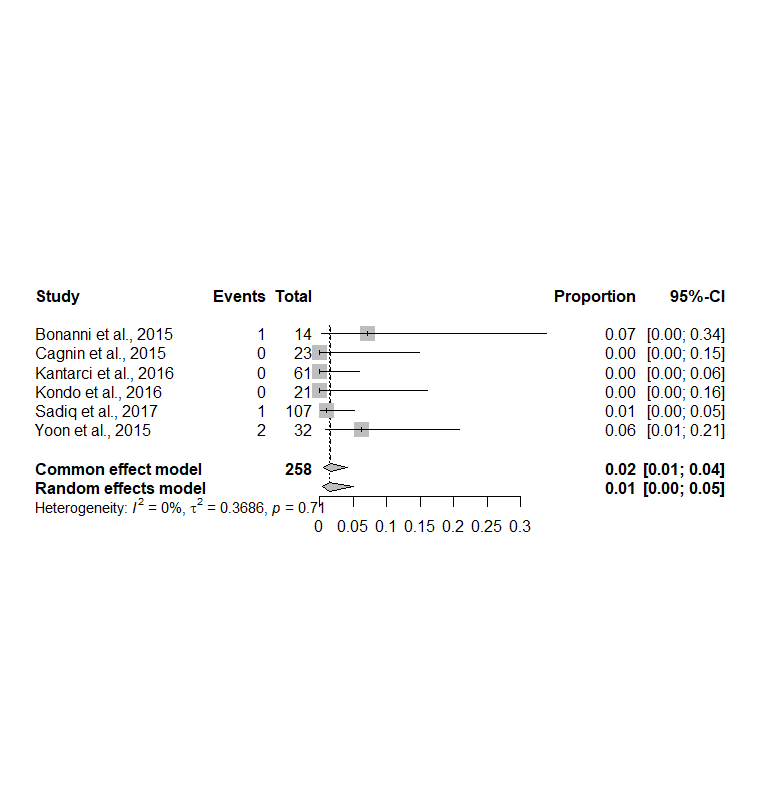


**Supplementary Figure 1C. Visual hallucinations: Stable MCI**
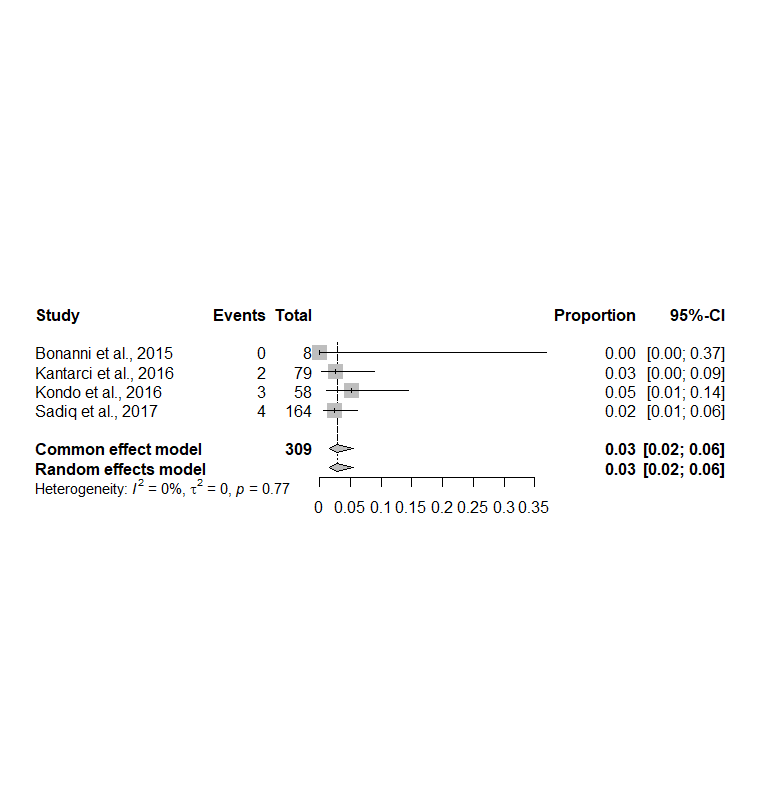


**Supplementary Figure 1D. Visual hallucinations: MCI-LB vs MCI-AD**


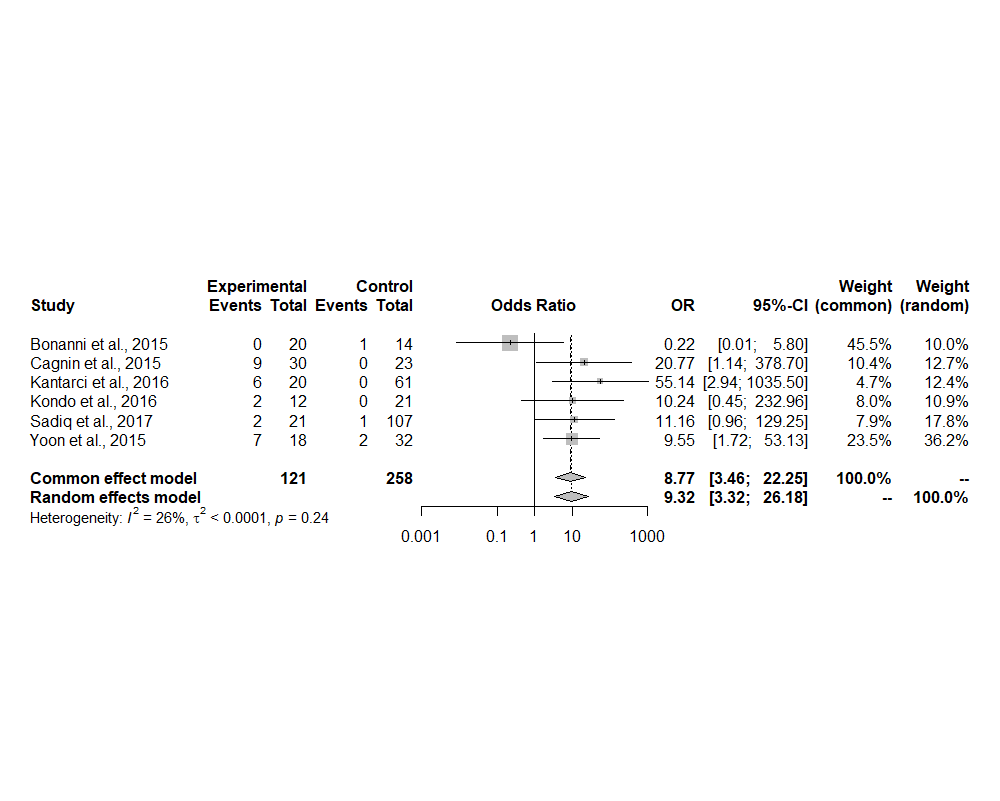


**Supplementary Figure 1E. Visual hallucinations: MCI-LB vs stable MCI**


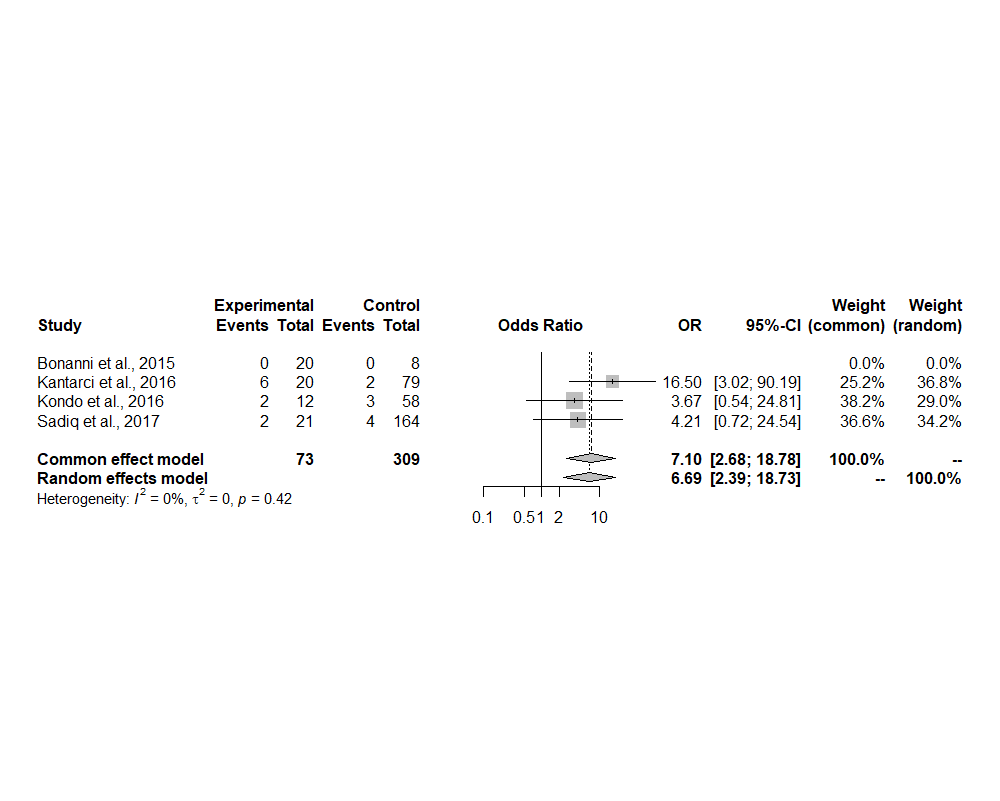


**Cognitive fluctuations**

**Supplementary Figure 2A. Cognitive Fluctuations: MCI-LB**


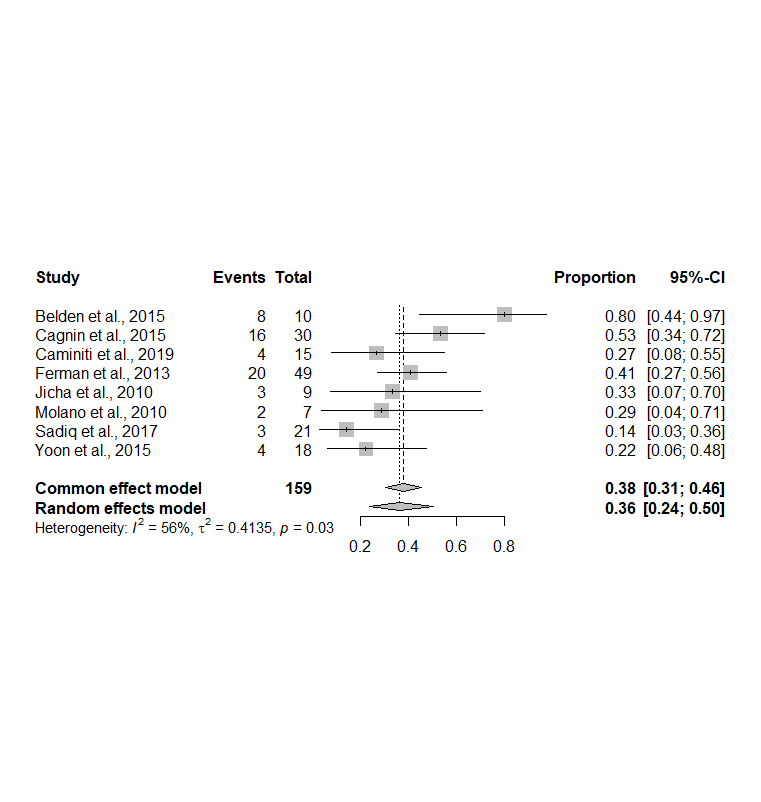


**Supplementary Figure 2B. Cognitive Fluctuations: MCI-AD**


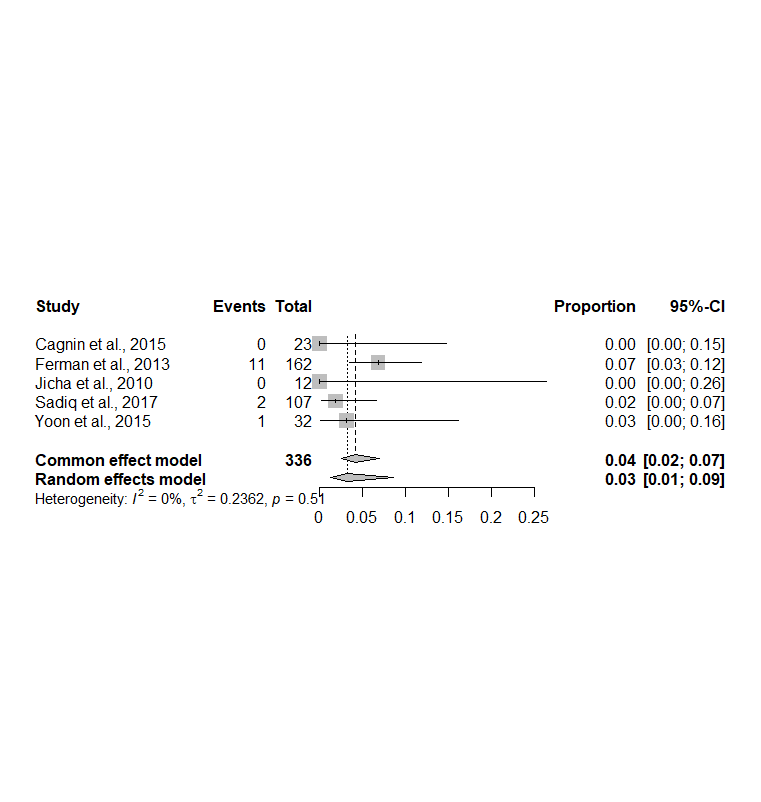


**Supplementary Figure 2C. Cognitive Fluctuations: MCI-LB vs MCI-AD**
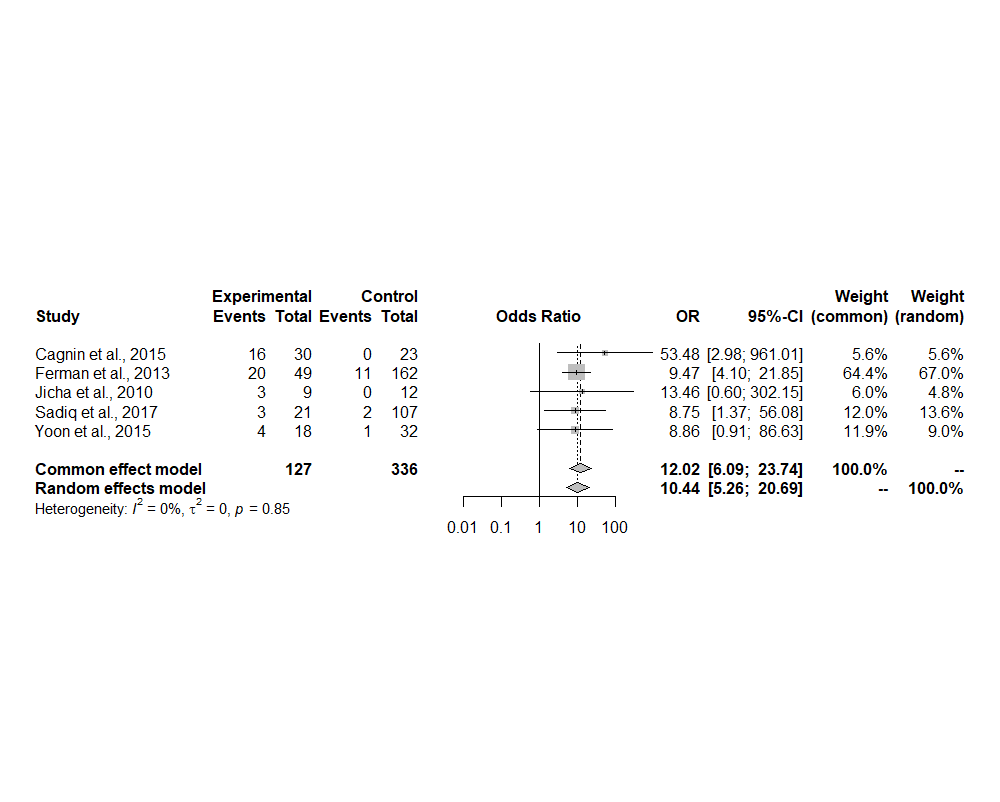


**Parkinsonism**

**Supplementary Figure 3A. Parkinsonism: MCI-LB**


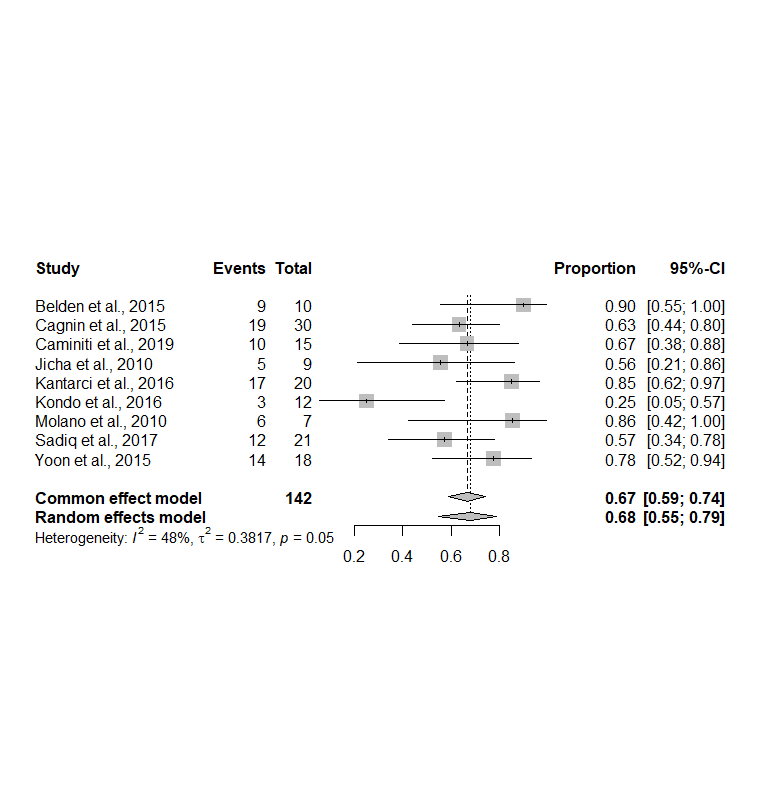


**Supplementary Figure 3B. Parkinsonism: MCI-AD**


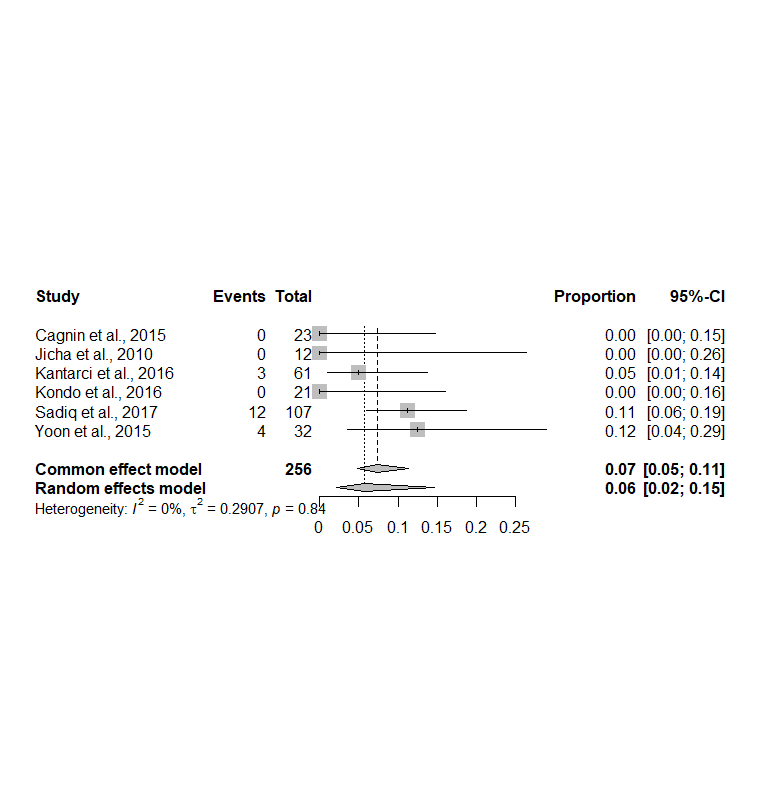


**Supplementary Figure 3C. Parkinsonism: Stable MCI**


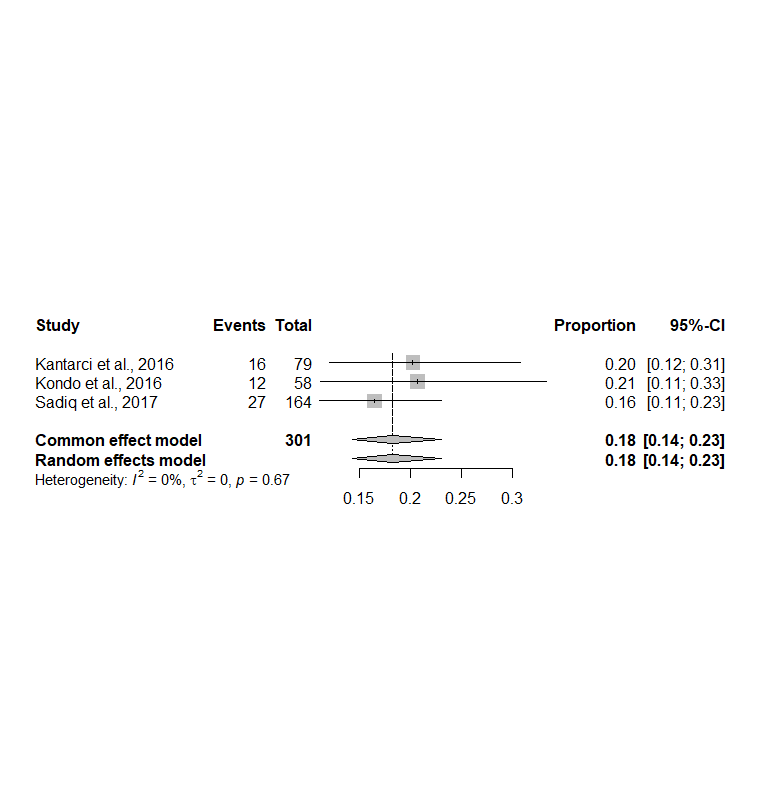


**Supplementary Figure 3D. Parkinsonism: MCI-LB vs MCI-AD**


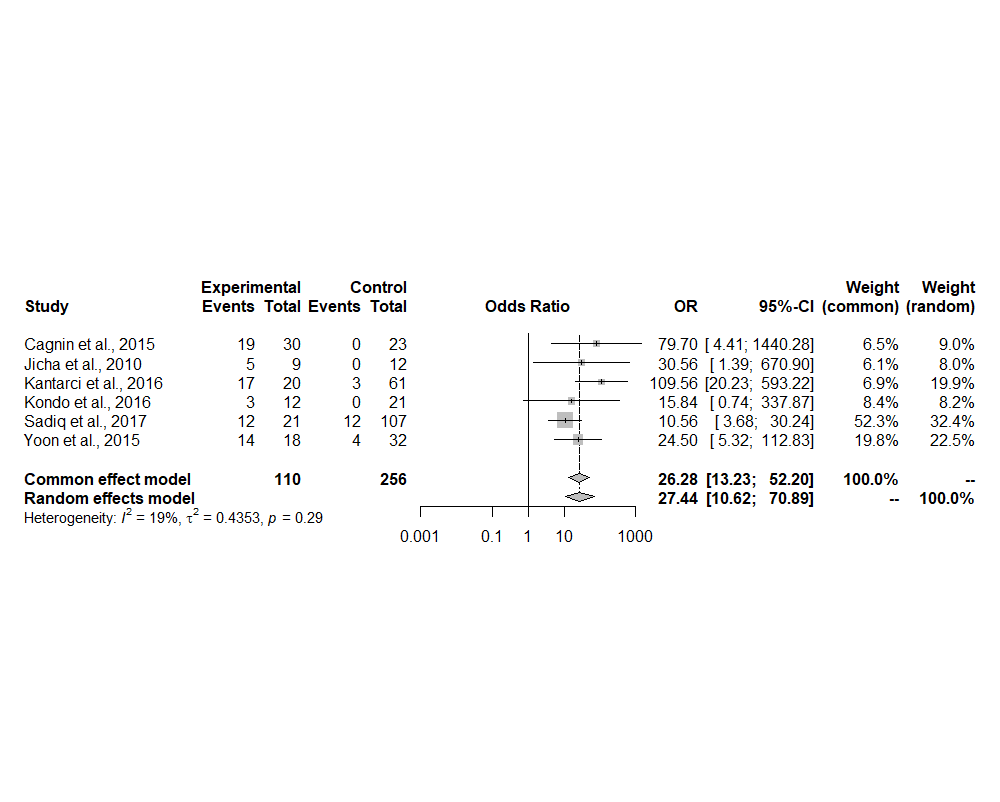


**Supplementary Figure 3E. Parkinsonism: MCI-LB vs stable MCI**


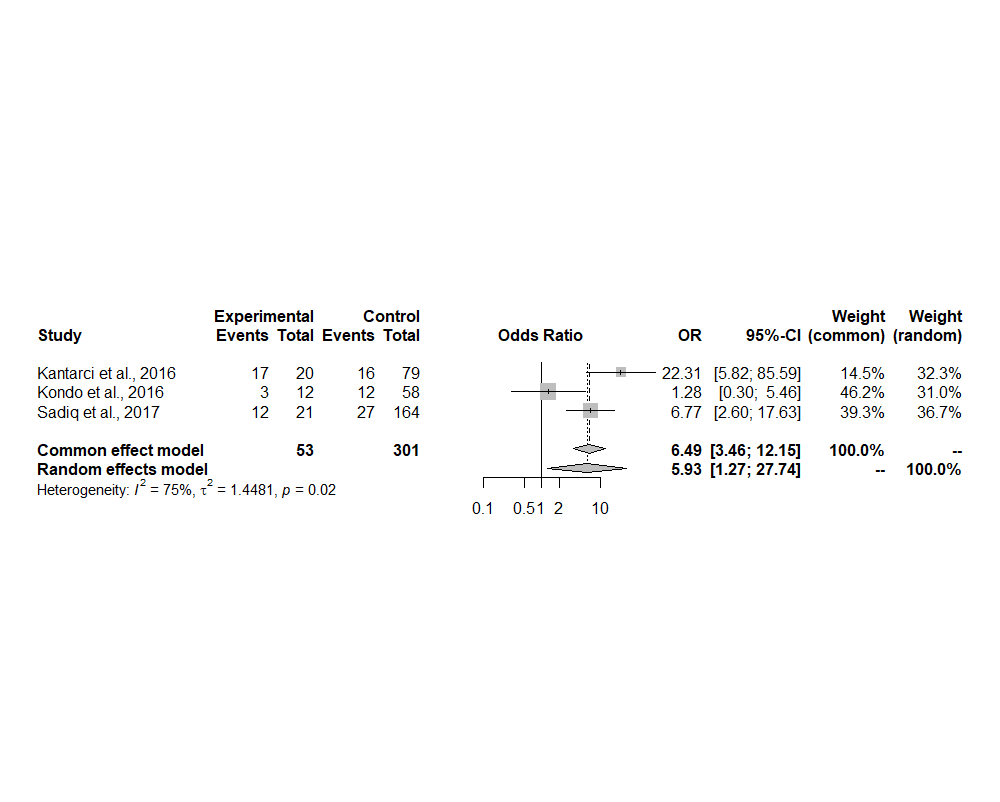


**REM-sleep behaviour disorder**

**Supplementary Figure 4A. REM-sleep behaviour disorder: MCI-LB**


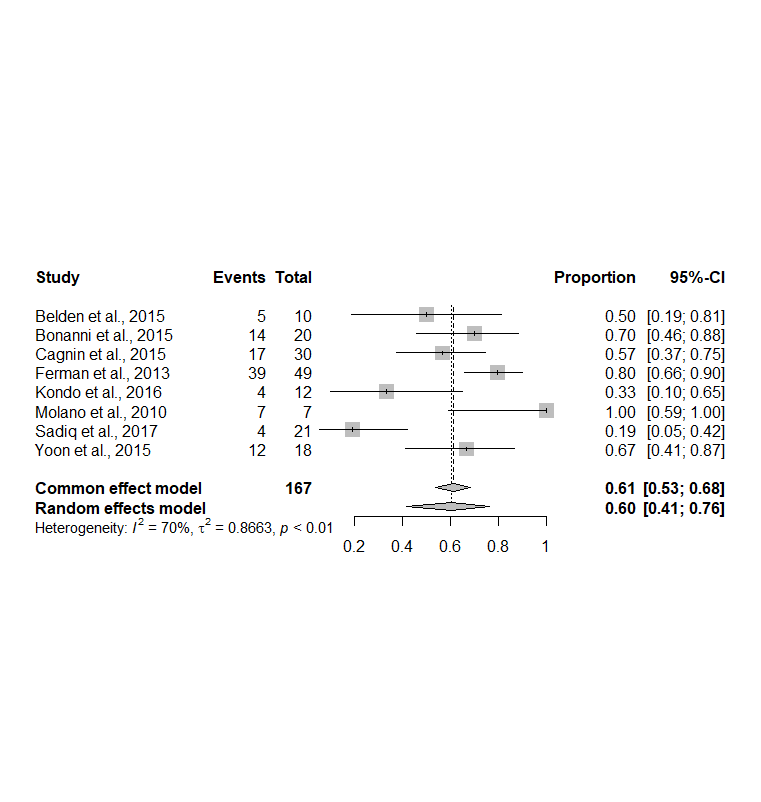


**Supplementary Figure 4B. REM-sleep behaviour disorder: MCI-AD**


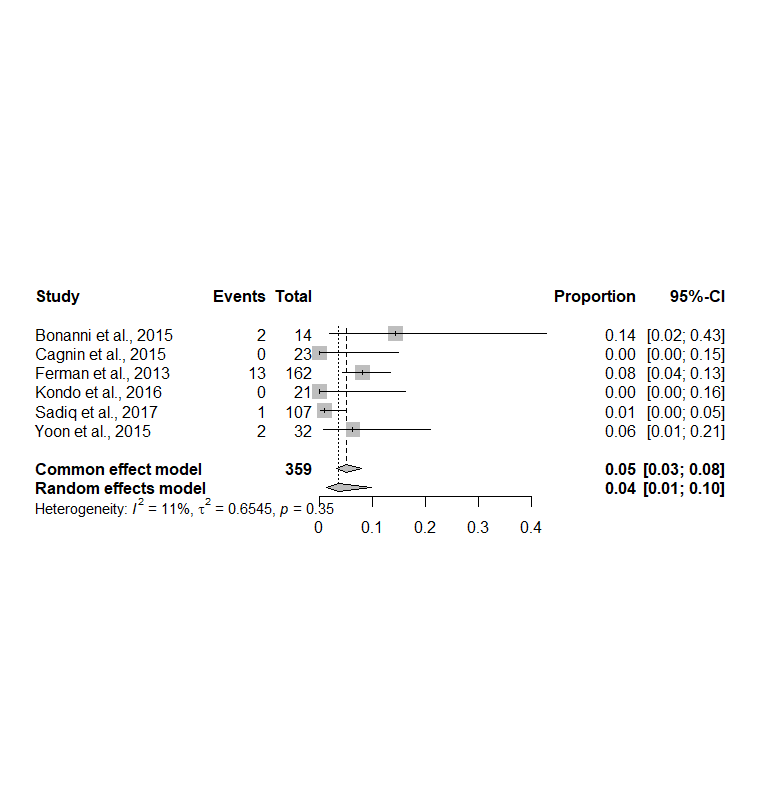


**Supplementary Figure 4C. REM-sleep behaviour disorder: Stable MCI**


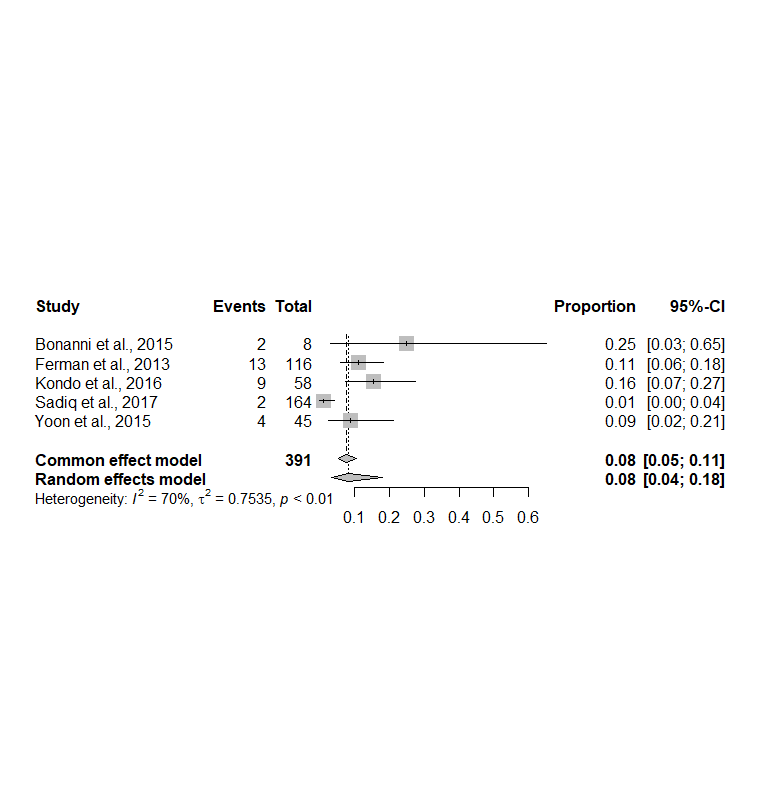


**Supplementary Figure 4D. REM-sleep behaviour disorder: MCI-LB vs MCI-AD**


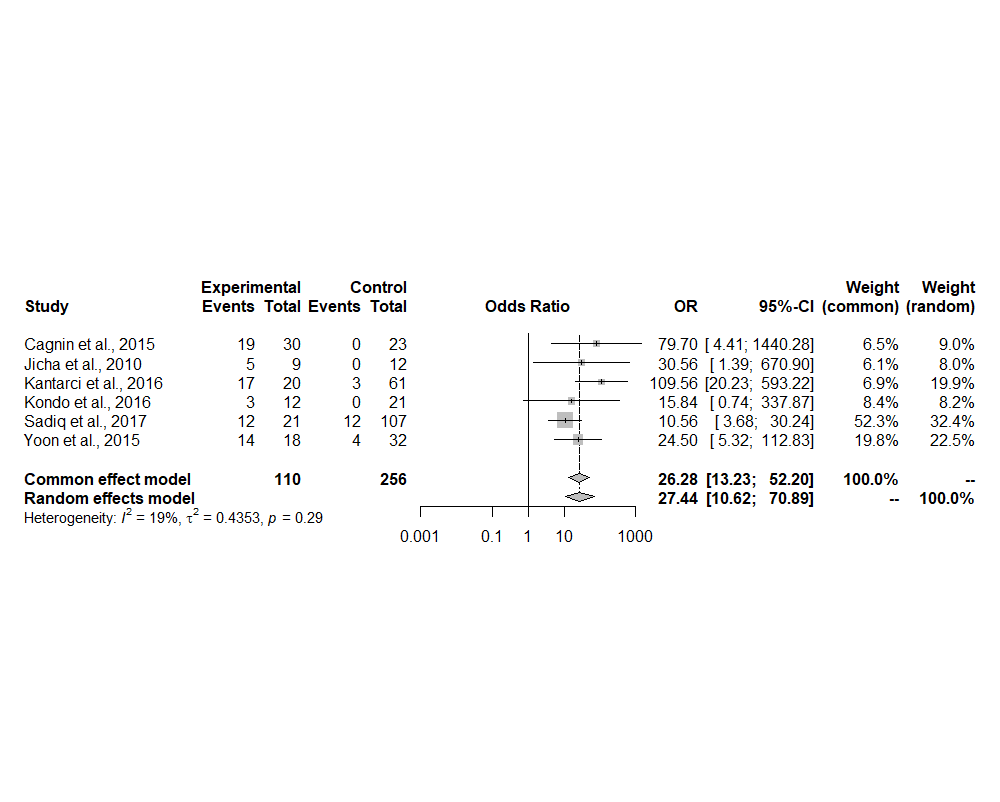


**Supplementary Figure 4E. REM-sleep behaviour disorder: MCI-LB vs stable MCI**


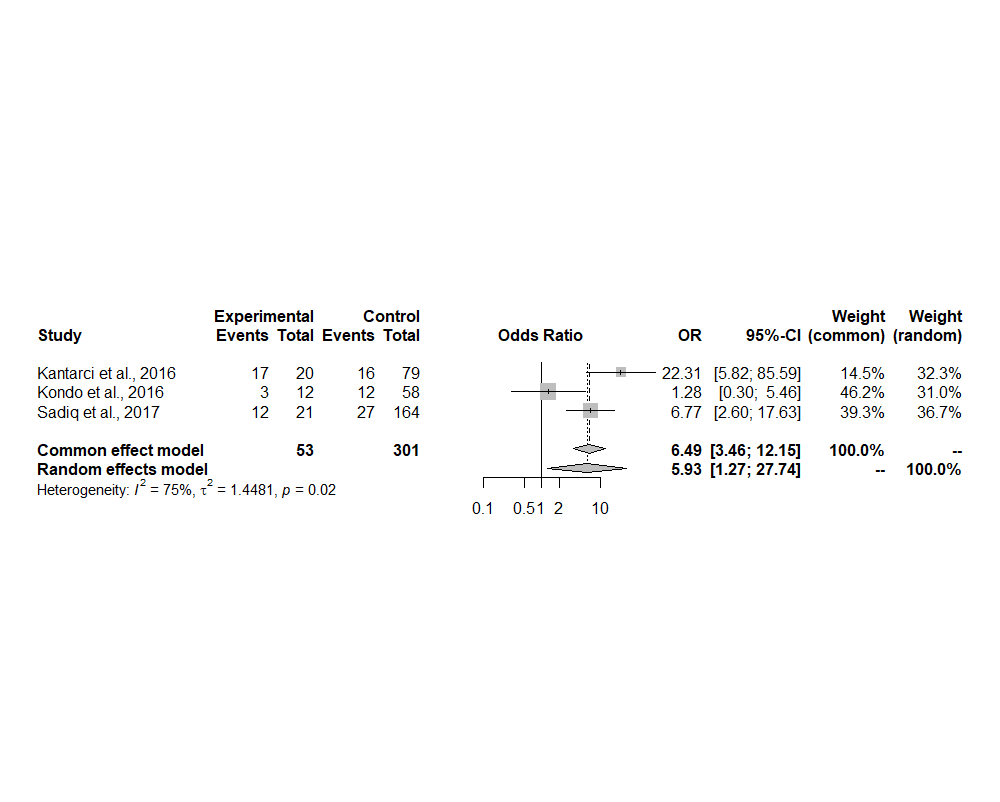


**Supportive clinical features**

**Anxiety**

**Supplementary Figure 5A. Anxiety: MCI-LB**


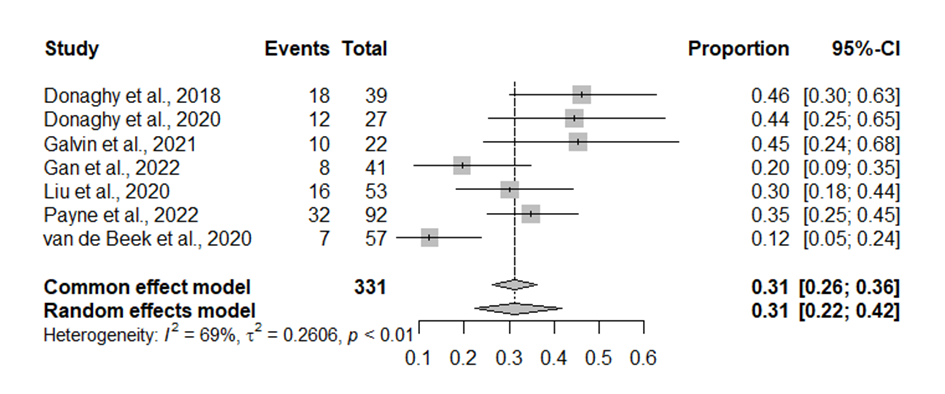


**Supplementary Figure 5B. Anxiety: MCI-AD**


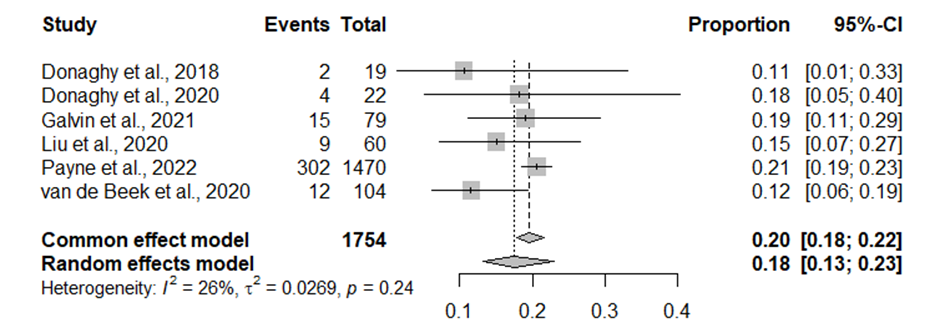


**Supplementary Figure 5C. Anxiety: MCI-LB vs MCI-AD**


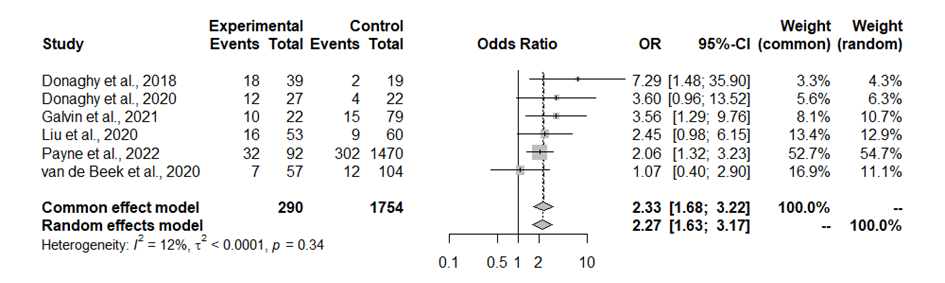


**Depression**

**Supplementary Figure 6A. Depression: MCI-LB**


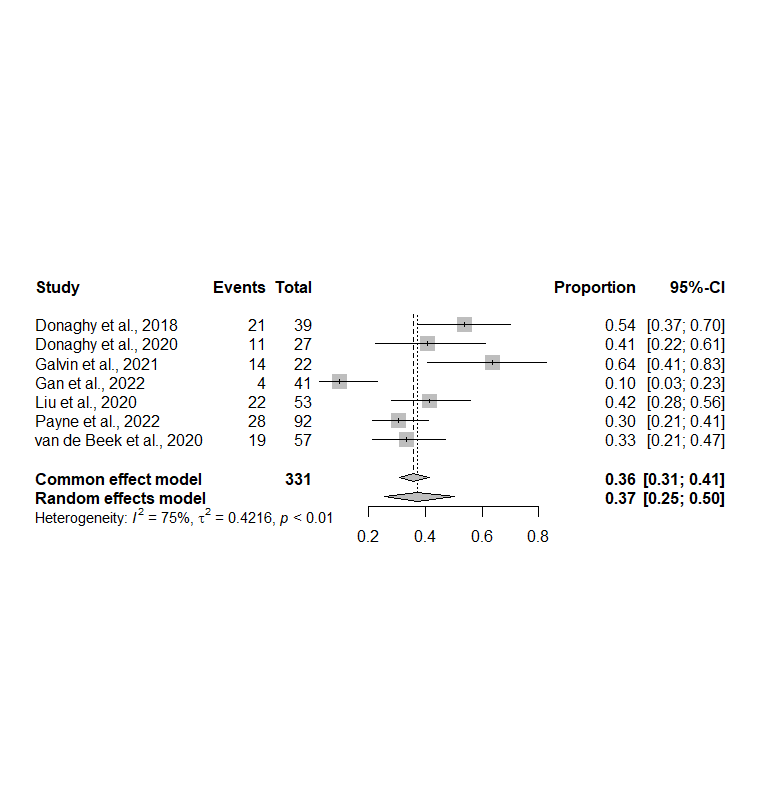


**Supplementary Figure 6B. Depression: MCI-AD**


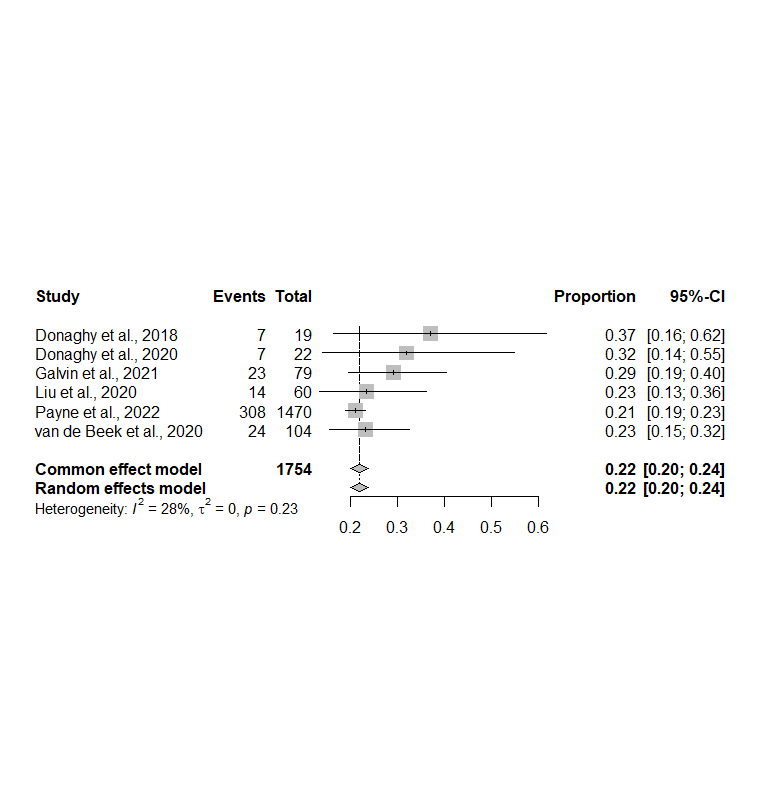


**Supplementary Figure 6C. Depression: MCI-LB vs MCI-AD**


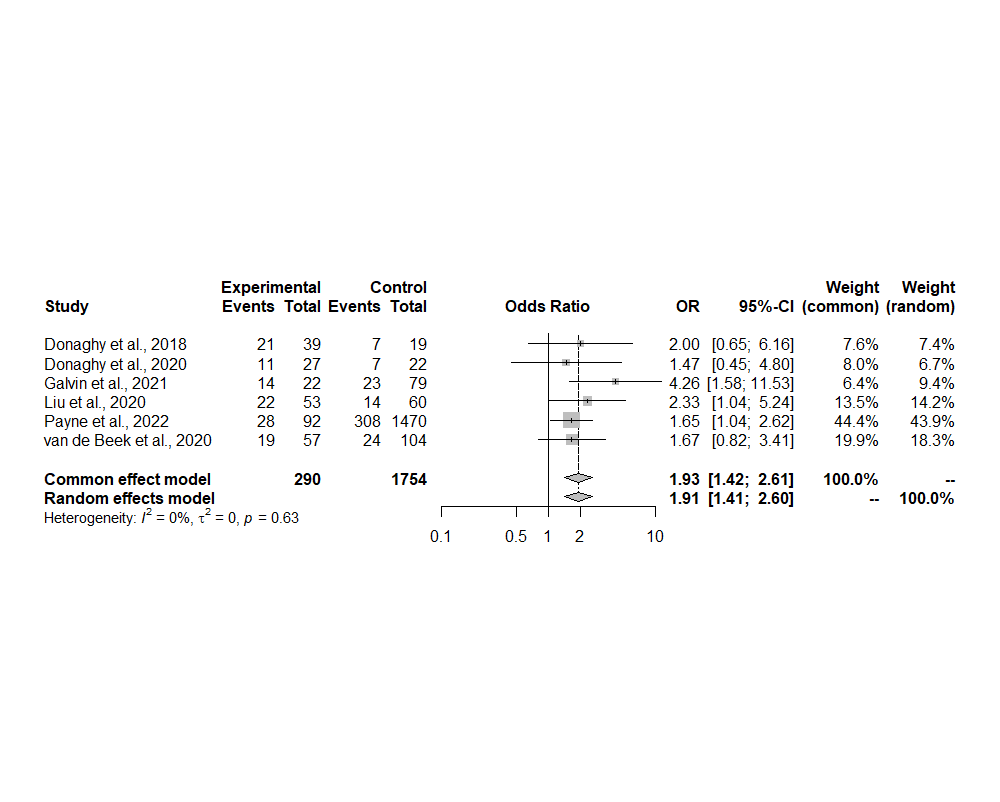


**Apathy**

**Supplementary Figure 7A. Apathy: MCI-LB**


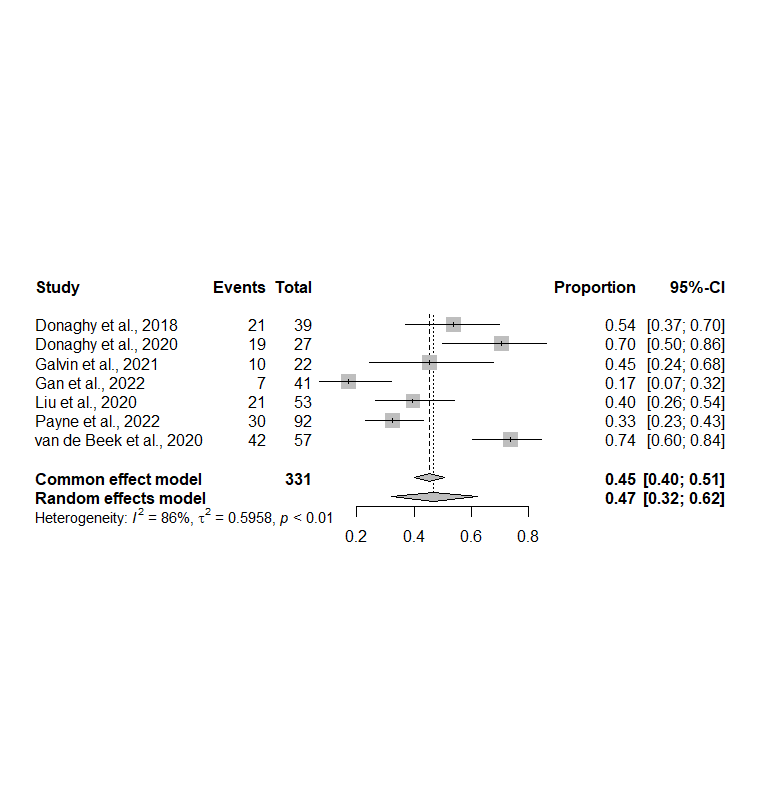


**Supplementary Figure 7B. Apathy: MCI-AD**


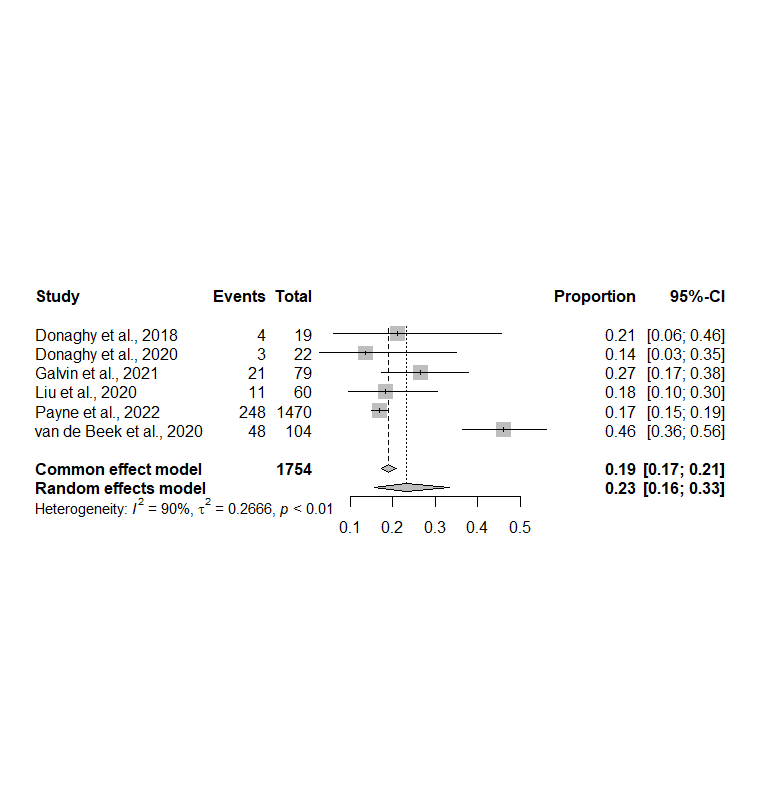


**Supplementary Figure 7C. Apathy: MCI-LB vs MCI-AD**


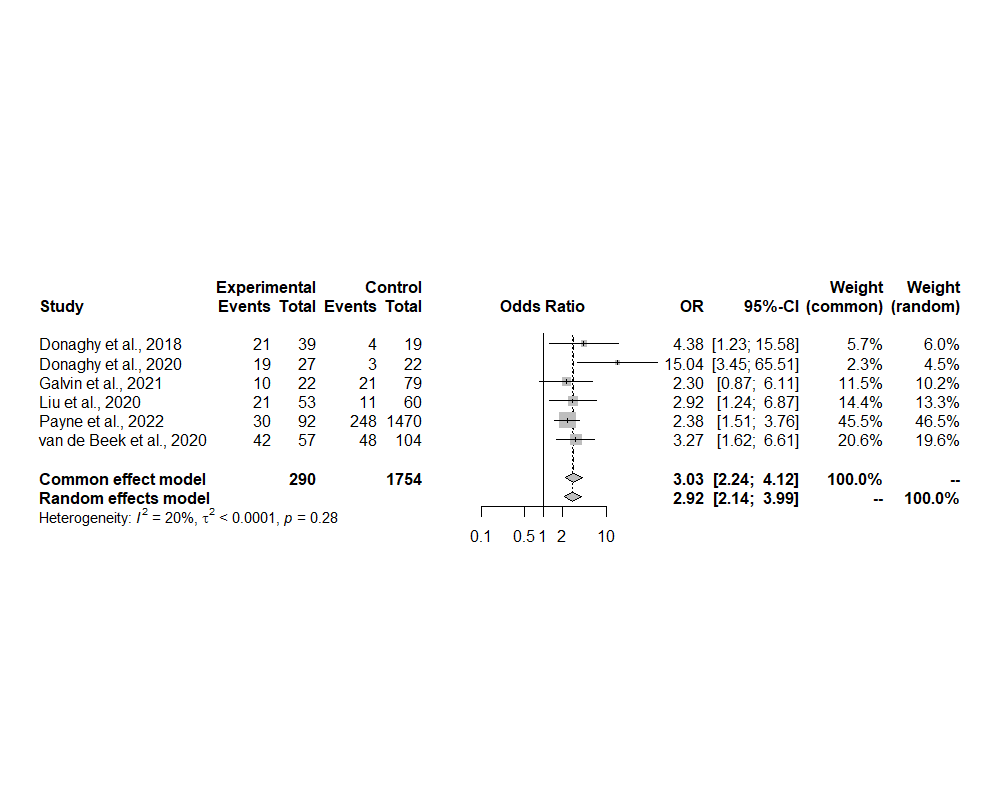


**Delusions**

**Supplementary Figure 8A. Delusions: MCI-LB**


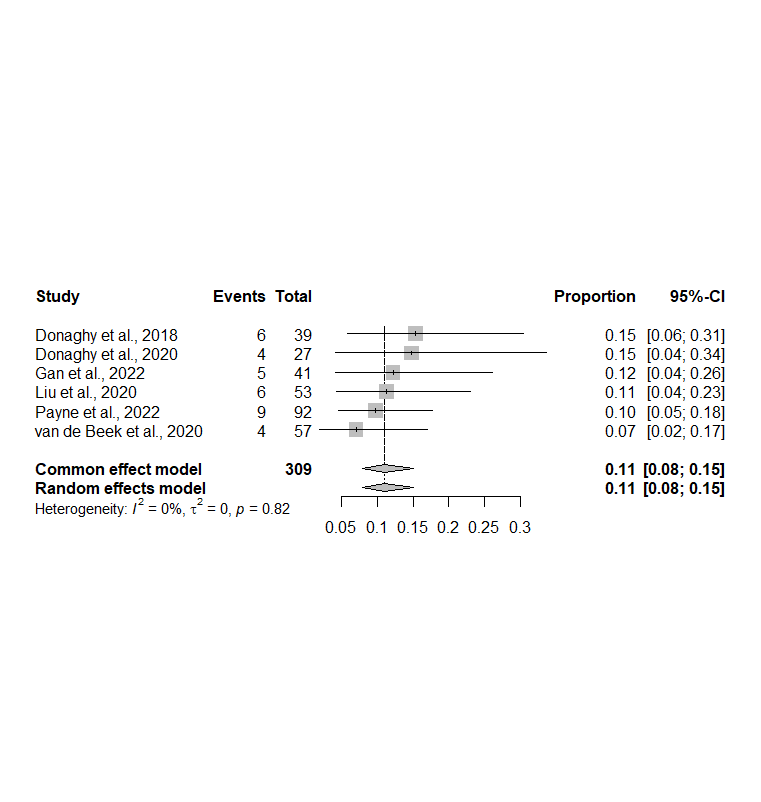


**Supplementary Figure 8B. Delusions: MCI-AD**


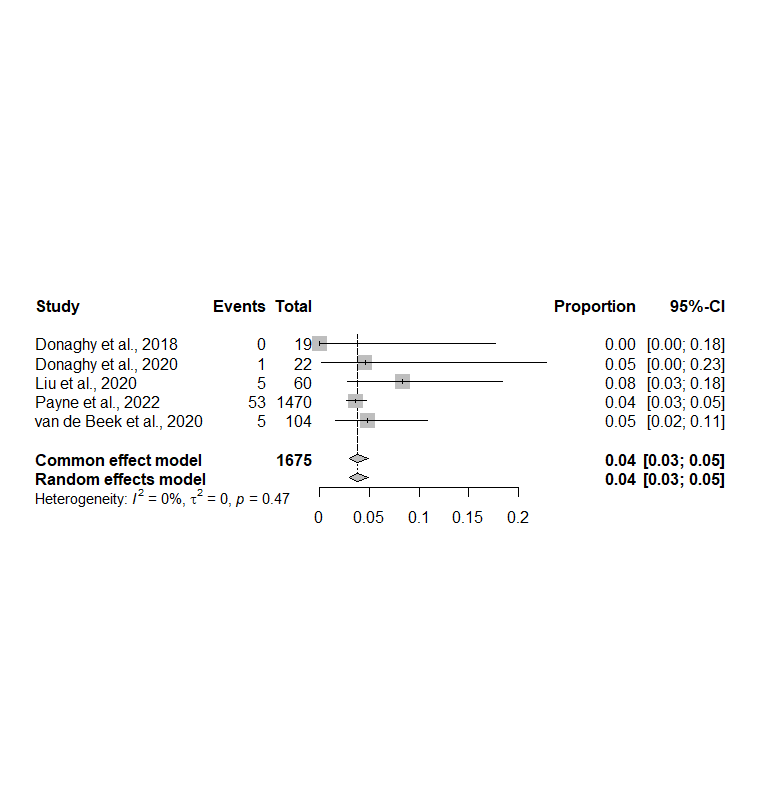


**Supplementary Figure 8C. Delusions: MCI-LB vs MCI-AD**


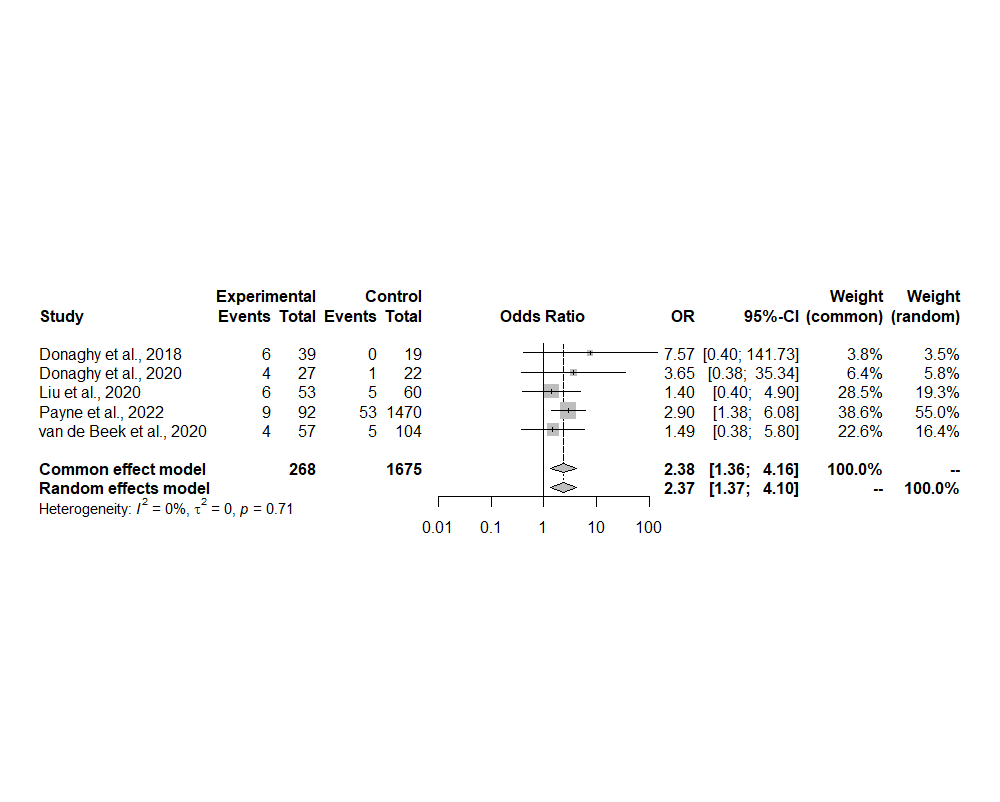


**Non-visual hallucinations**

**Supplementary Figure 9A. Non-visual hallucinations: MCI-LB**


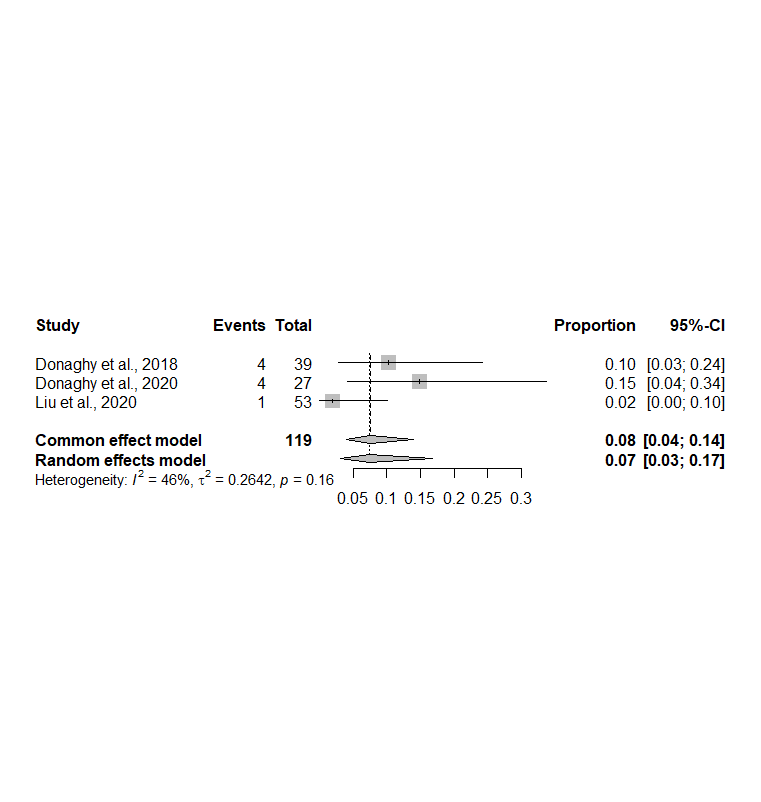


**Supplementary Figure 9B. Non-visual hallucinations: MCI-AD**


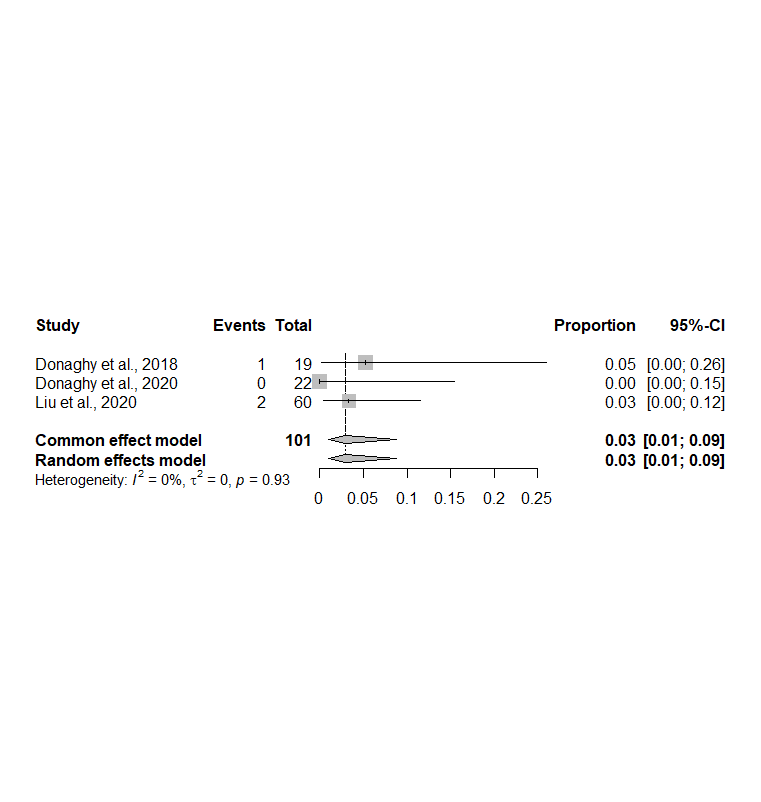


**Supplementary Figure 9C. Non-visual hallucinations: MCI-LB vs MCI-AD**


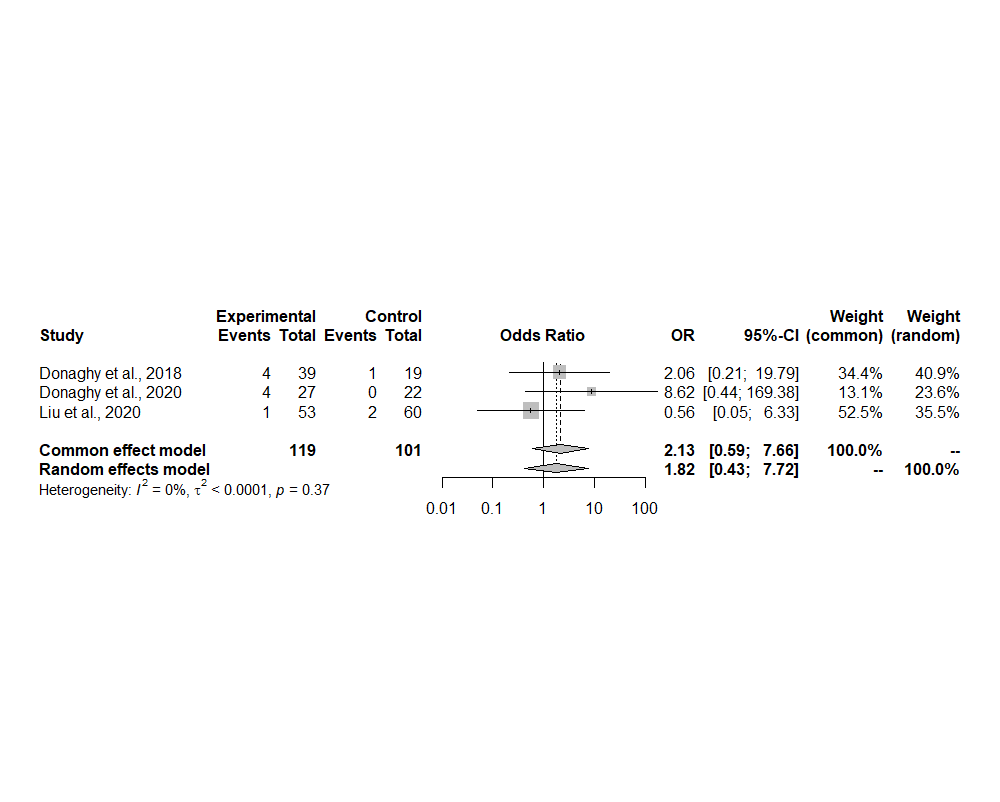


**Constipation**

**Supplementary Figure 10A. Constipation: MCI-LB**


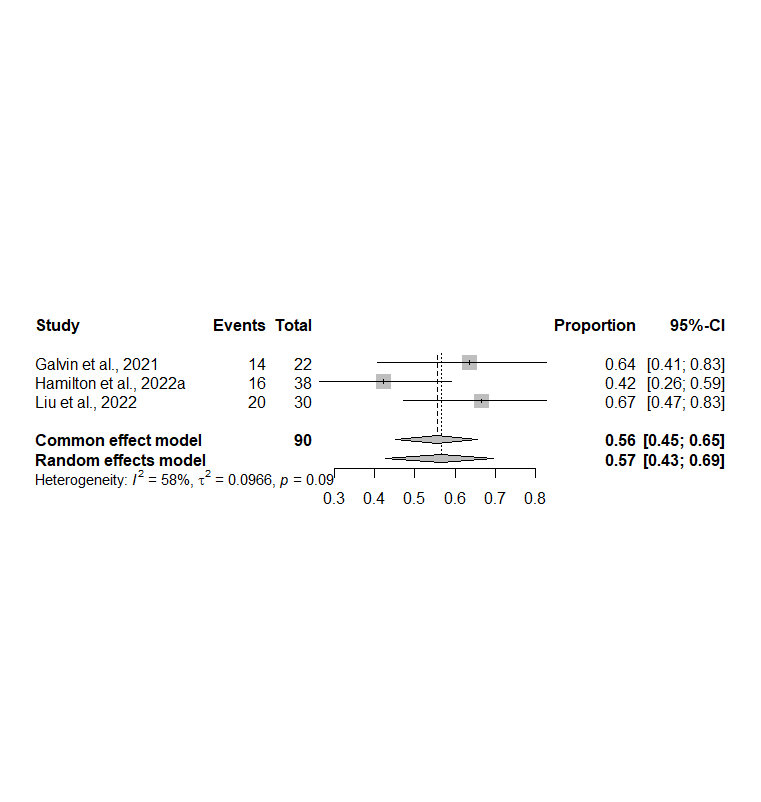


**Supplementary Figure 10B. Constipation: MCI-AD**


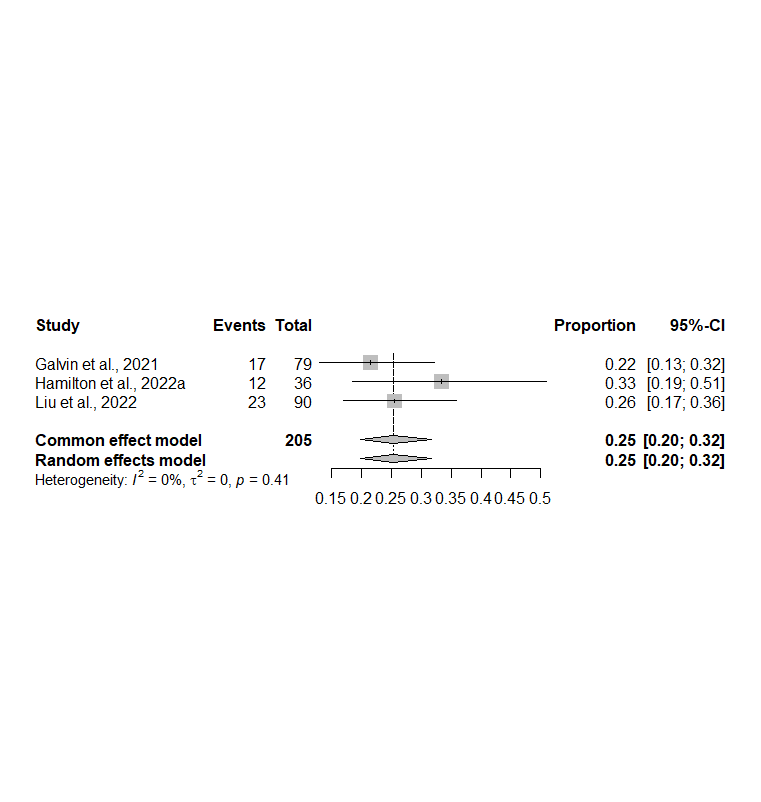


**Supplementary Figure 10C. Constipation: MCI-LB vs MCI-AD**


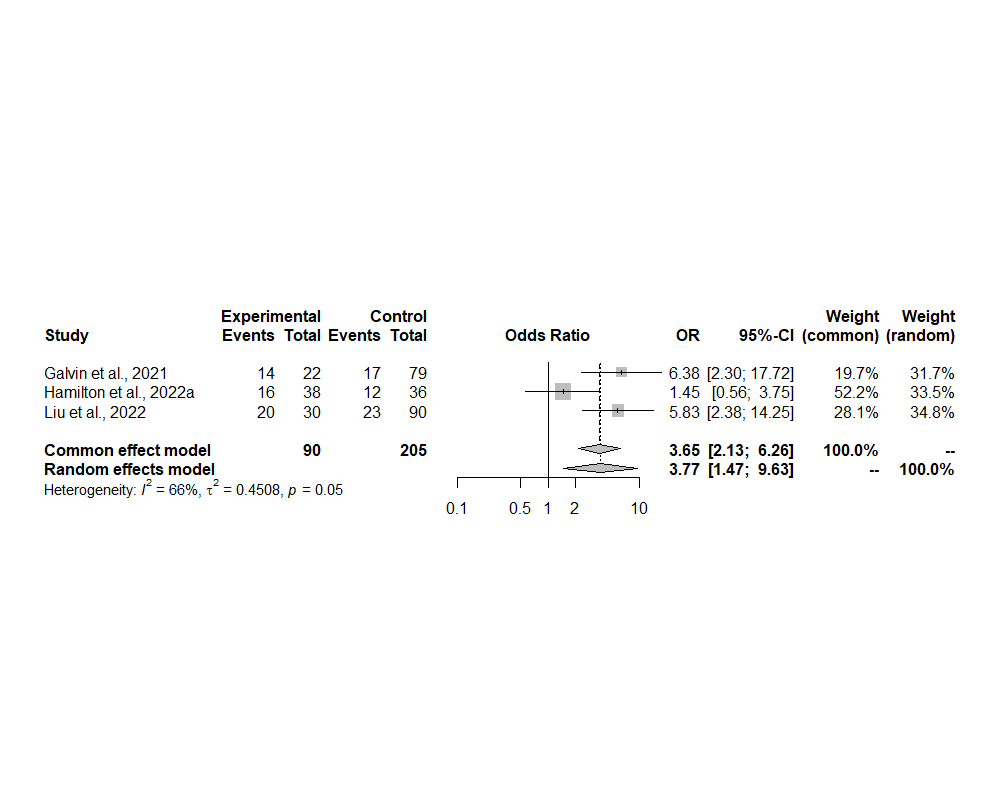


**Difficulty emptying bladder**

**Supplementary Figure 11A. Difficulty emptying bladder: MCI-LB**


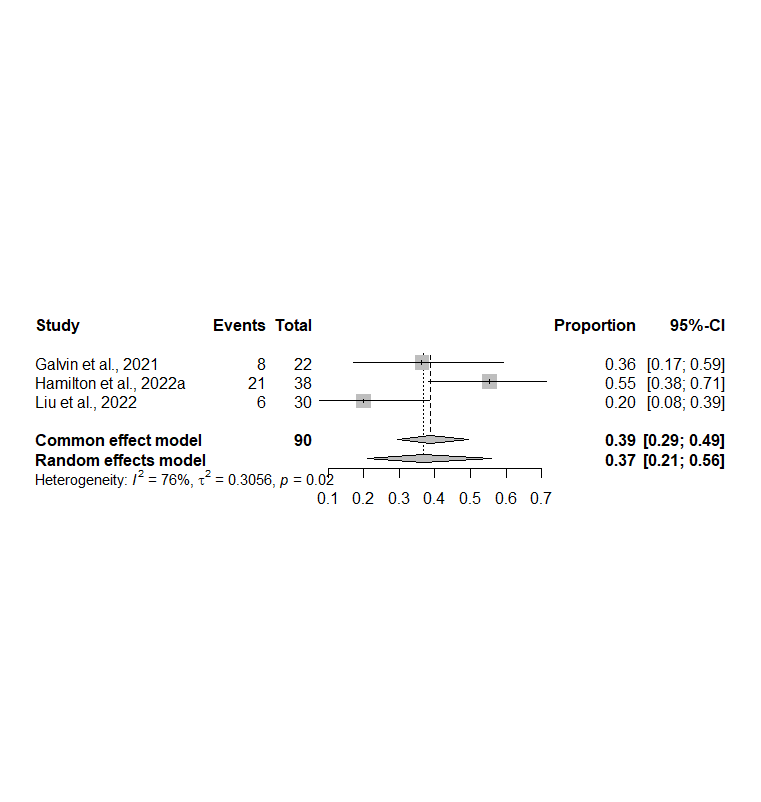


**Supplementary Figure 11B. Difficulty emptying bladder: MCI-AD**


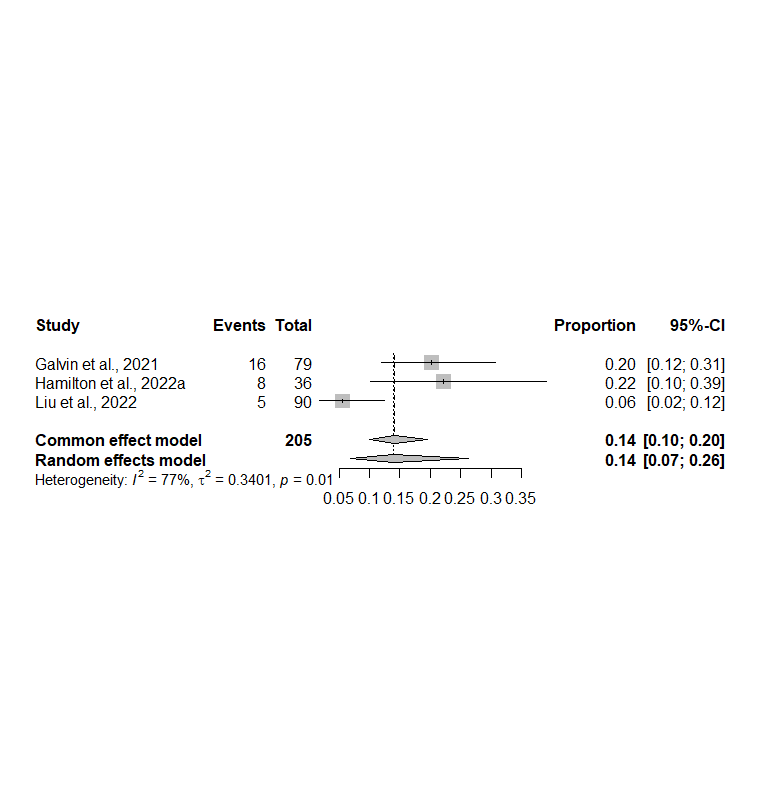


**Supplementary Figure 11C. Difficulty emptying bladder: MCI-LB vs MCI-AD**


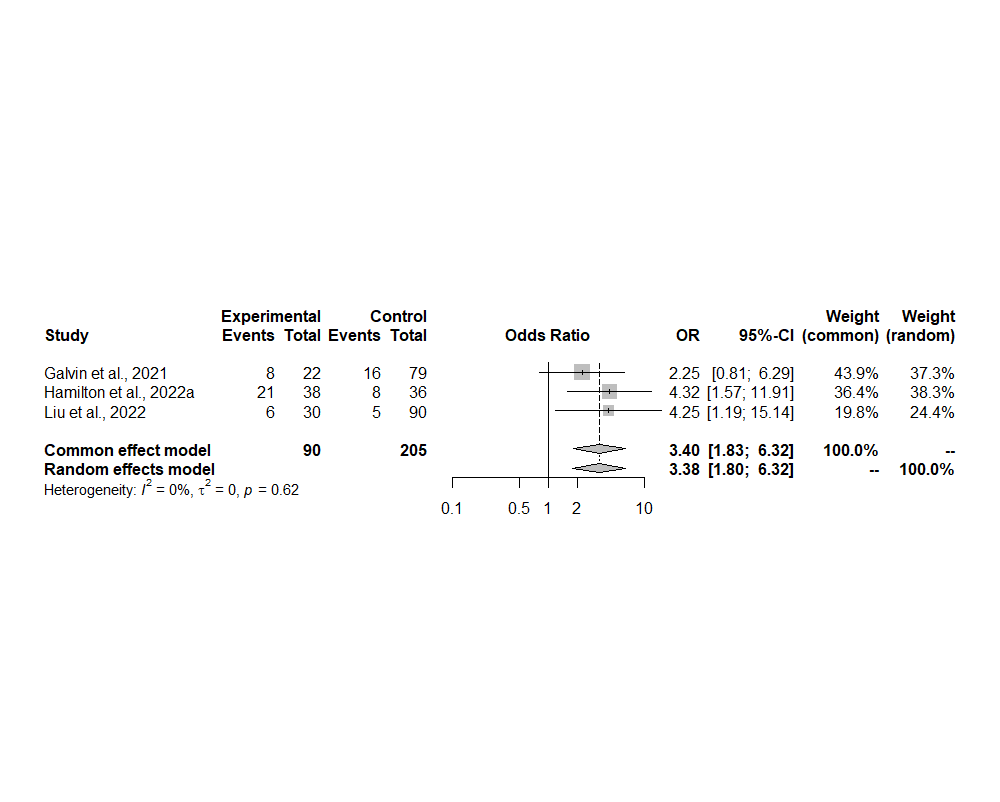

Supplement: Supplementary Figures [file NIHMS1944285-supplement-Supplementary_Figures.docx]
