# Supplementary material for: Research diagnostic criteria for mild cognitive impairment with Lewy bodies: A systematic review and meta-analysis
Source: Alzheimers Dement. Author manuscript; Available in PMC 2023 Dec 4. (PMC10695683; doi:10.1002/alz.13105)
Supplement: Supplementary Tables [file NIHMS1944285-supplement-Supplementary_Tables.pdf]

Supplementary Table 1. Core clinical features

Author, year<br><br>Method of diagnostic classification	Study sample	Symptom Prevalence			
		Symptom	Prob MCI-LB %	MCI-AD %	Stable MCI %
Jicha 2010 [1]<br><br>Post Mortem	MCI-LB n=9<br>AD MCI n=12	Fluctuations<br>Parkinsonism	33<br>56	0<br>0	-<br>-
Molano 2010* [2]<br><br>Post Mortem	MCI-LB n=8	Fluctuations<br>Hallucinations<br>Parkinsonism<br>RBD	29<br>63<br>75<br>88	-<br>-<br>-<br>-	-<br>-<br>-<br>-
Belden, 2014 [3]<br><br>Clinical (DLB)	Prob MCI-LB n=10	Fluctuations<br>Hallucinations<br>Parkinsonism<br>RBD	80<br>50<br>90<br>50	-<br>-<br>-<br>-	-<br>-<br>-<br>-
Bonanni 2015 [4]<br><br>Clinical (DLB)	Prob MCI-LB n=20<br>AD MCI n=14<br>Stable MCI n=8	Hallucinations<br>RBD	0<br>70	7<br>14	0<br>25
Cagnin, 2015a (Cagnin 2015b) [5, 6]<br><br>Clinical (DLB)	Prob MCI-LB n=30<br>AD-MCI n=23	Fluctuations<br>Hallucinations<br>Parkinsonism<br>RBD	53<br>30<br>63<br>57	0<br>0<br>0<br>0	-<br>-<br>-<br>-
Caminiti 2019 [7]<br><br>Clinical (DLB)	Prob MCI-LB n=15	Fluctuations<br>Hallucination<br>Parkinsonism<br>RBD	27<br>53<br>67<br>-	-<br>-<br>-<br>-	-<br>-<br>-<br>-
Ferman, 2013† [8]<br><br>Clinical (DLB)	Prob MCI-LB n=49<br>AD MCI n=162<br>Stable MCI n=116	Fluctuations<br>RBD	41<br>80	7<br>8	9<br>11
Kantarci 2016† [9]<br><br>Clinical (DLB)	Prob MCI-LB n=20<br>AD MCI n=61<br>Stable MCI n=79	Fluctuations<br>Hallucinations<br>Parkinsonism<br>RBD	40<br>30<br>85<br>85	0<br>0<br>5<br>7	6<br>3<br>20<br>23
Kantarci 2021† [10]<br><br>Clinical (DLB)	Prob MCI-LB n=17<br>AD MCI n=41	Fluctuations<br>Hallucinations<br>Parkinsonism<br>RBD	41<br>35<br>82<br>88	0<br>0<br>7<br>12	-<br>-<br>-<br>-
Kondo 2016 [11]<br><br>Clinical (DLB)	Prob MCI-LB n=12<br>AD MCI n=21<br>Stable MCI n=58	Hallucinations<br>Parkinsonism<br>RBD	17<br>25<br>33	0<br>0<br>0	5<br>21<br>16
Sadiq 2017 [12]<br><br>Clinical (DLB)	Prob MCI-LB n=21<br>AD MCI n=107<br>Stable MCI n=164	Fluctuations<br>Hallucinations<br>Parkinsonism<br>RBD	14<br>10<br>57<br>19	2<br>1<br>11<br>1	2<br>2<br>17<br>1

Yoon 2015 and Kim 2018 [13, 14]<br><br>Clinical (DLB)	Prob MCI-LB n=18<br>AD MCI n=32<br>Stable MCI n=45	Fluctuations<br>Hallucination<br>Parkinsonism<br>RBD	22<br>39<br>78<br>67	3<br>6<br>13<br>6	-<br>-<br>-<br>9
Hamilton 2021a<br>Hamilton 2021b [15, 16]<br><br>Clinical (DLB)	Prob MCI-LB n=61<br>Poss MCI-LB n=17<br>AD MCI n=33	Probable MCI-LB associated with later diagnosis of DLB, compared with MCI-AD. Increased number of diagnostic features (core clinical or proposed biomarkers) was associated with greater risk of dementia (HR 1.3 (1.1-1.6)). Fluctuations were associated with greater risk of dementia (HR 4.0 (2.0-7.8)).			
van de Beek 2020 [17]<br><br>Clinical (DLB)	Prob MCI-LB n=73<br>AD MCI n=124	The number of core clinical features did not predict time to progression to dementia within MCI-LB (HR 1.1 (0.8-1.5)).			

AD Alzheimer's disease; HR hazard ratio; MCI-LB mild cognitive impairment with Lewy bodies; Prob probable; Poss possible; RBD REM sleep behaviour disorder.
Stable MCI refers to those that remain MCI and those that revert to normal cognition.
†=there may be overlap in cases in these cohorts.
*=only 7 subjects with complete data included in meta-analysis.
Method of diagnostic classification: Post Mortem – post mortem confirmation of Lewy body pathology; Clinical (DLB) – clinical diagnosis of DLB after conversion to dementia, data reported are from MCI phase.
Articles listed with post-mortem studies first, then in alphabetical order by first author.

Supplementary Table 2. Supportive Clinical Features

Author, year<br><br>Method of diagnostic classification	Study sample	Symptom	Prob MCI-LB %	MCI-AD %
Donaghy 2017 [18]<br><br>Clinical (MCI-LB)	Prob MCI-LB=36<br>MCI-AD=21	Drooling	53	10
		Frequent falls	43	11
		Loss of smell	44	19
		Daytime sleepiness	56	29
		Transient loss of consciousness	0	3
		Dizziness/fainting	47	29
		Sensitivity heat/cold	64	52
		Double vision	18	10
Donaghy 2018 [19]<br><br>Clinical (MCI-LB/DLB)	Prob MCI-LB=39<br>MCI-AD=19	Anxiety	46	11
		Apathy	54	21
		Delusions	15	0
		Non-visual hallucinations	10	5
		Depression	54	37
		MCI-LB showed a relative drop in postural blood pressure compared with MCI-AD		
Donaghy 2022 [20]<br><br>(Clinical (MCI-LB/DLB)	Prob MCI-LB=27<br>MCI-AD=22	Apathy	70	14
		Delusions	15	5
		Non-visual hallucinations	15	0
		Depression	41	32
		Anxiety	44	18
Durcan 2019 [21]<br><br>Clinical (MCI-LB)	Prob/poss MCI-LB=48<br>MCI-AD=27	Gastroparesis	6	0
Gan 2022 [22]<br><br>Clinical (MCI-LB)	N=41 MCI-LB<br>N=48 controls<br>Al controls scored zero for features	Apathy	17	-
		Delusions	12	-
		Depression	10	-
		Anxiety	20	-
Galvin 2021 [23]<br><br>Clinical (MCI-LB)	MCI-LB=22<br>MCI-AD=79	Depression	64	29
		Anxiety	47	19
		Apathy	47	27
		Dysphagia	19	5
		Decreased libido	43	22
		Decrease sexual performance	52	25
		Double vision	19	3
		Constipation	62	21
		Obstipation	43	15
		Incomplete bladder emptying	38	20
		Lightheaded change in position	33	17
		Lightheaded prolonged stand	19	7

		Fainting	14	3
		Daytime sleepiness	67	38
		Orthostatic hypotension	13	2
		Number of autonomic features greater in MCI-LB 5.6 (SD 3.2) than MCI-AD 3.1 (2.5), p<0.001.		
Hamilton 2022 [24]<br><br>Clinical (MCI-LB/DLB)	38 prob MCI-LB<br>19 Poss MCI-LB<br>36 MCI-AD<br>33 control	Orthostatic intolerance	42	22
		Vasomotor skin changes	16	11
		Abnormal sweating	29	20
		Dry eyes	18	19
		Dry mouth	40	22
		Decreased appetite	34	44
		Bloating	32	19
		Vomiting	8	6
		Cramping abdominal pain	24	14
		Diarrhoea	34	31
		Constipation	42	33
		Loss of bladder control	45	17
		Difficulty passing urine	21	8
		Difficulty emptying bladder	55	22
		Sensitivity to bright light	34	22
		Trouble focusing eyes	26	17
Hamilton 2022 [25]<br><br>Clinical (MCI-LB/DLB)	Prob MCI-LB n=37<br>Poss. MCI-LB n=17<br>MCI-AD n=35	Prob MCI-LB were more likely to demonstrate an abnormal blood pressure response to the Valsalva manoevre – drop in BP that did not recover after 10s (44% v 11%). No difference between the groups in abnormal heart rate on Valsalva manoevre (21% v 14%), or orthostatic hypotension (19% v 18%). Possible MCI were more likely to demonstrate orthostatic hypotension than MCI-AD (47% v 18%).		
Kim 2018 [14]<br><br>Clinical (DLB)	Prob MCI-LB= 23<br>MCI-AD=32	MCI-LB group exhibited significantly lower levels of almost all heart rate variability parameters compared to MCI-AD patients.		
Liu 2021 [26]<br><br>Clinical (MCI-LB)	Prob MCI-LB=53<br>MCI-AD=60	Anxiety	30	15
		Apathy	40	18
		Depression	42	23
		Non-visual halls	2	2
		Delusions	11	8
Liu 2022 [27]<br><br>Clinical (MCI-LB)	Cross-sectional<br>30 Prob MCI-LB<br>90 MCI-AD	Gastrointestinal (any)	73	36
		Dysphagia	17	10
		Salivation	30	7
		Swallowing/choking	3	0
		Abdominal fullness	13	8
		Constipation	67	26
		Loose stool	0	1
		Fecal incontinence	3	1

		Urinary (any)	47	28
		Urgency	13	10
		Incontinence	17	3
		Incomplete emptying	20	6
		Weak stream	13	4
		Frequency	37	21
		Nocturia	33	12
		Cardiovascular (any)	27	7
		Lightheadedness (standing up)	27	7
		Lightheadedness standing for some time	13	0
		Syncope	0	0
		Thermoregulatory (any)	33	7
		Hyperhidrosis (day)	20	3
		Hyperhidrosis (night)	13	1
		Cold intolerance	7	2
		Heat intolerance	7	1
		Oversensitivity to bright light	3	1
		Sexual dysfunction	20	7
Payne 2022 [28]<br><br>Clinical (DLB)	Longitudinal<br><br>92 Prob MCI-LB<br>1470 MCI-AD	Apathy	33	17
		Delusions	10	4
		Depression	30	21
		Anxiety	35	21
Thomas 2022 [29]<br><br>Clinical MCI-LB/DLB)	38 Prob MCI-LB<br>19 Poss MCI-LB<br>33 MCI-AD<br>32 Healthy controls	Olfactory function measured by 'Sniffin' Sticks' was worse in prob MCI-LB than MCI-AD, poss MCI-LB and controls. The AUROC for prob MCI-LB v MCI-AD was 0.67.		
Van de Beek 2020 [17]<br><br>Clinical (MCI-LB/DLB)	Prob MCI-LB=57<br>MCI-AD=104	Delusions	7	5
		Depression	33	23
		Anxiety	12	12
		Apathy	74	46
		Postural hypotension	43	26
Yoon 2015 [13]<br><br>Clinical (DLB)	Prob MCI-LB=18<br>MCI-AD=32<br>Stable-MCI=45	Olfaction – Cross Cultural Smell Index was significantly lower in MCI-LB (4.6; 95% CI: 4.0–5.3) than MCI-AD (6.4; 95% CI: 6.0–6.7, p<0.001) or stable MCI (7.3; 95% CI: 6.9–7.8, p<0.001). AUROC MCI-LB v MCI-AD 0.85 (95% CI: 0.72-0.97)		

AD Alzheimer's disease; HR hazard ratio; MCI-LB mild cognitive impairment with Lewy bodies; Prob probable; Poss possible; RBD REM sleep behaviour disorder
Stable MCI refers to those that remain MCI and those that revert to normal cognition.
Method of diagnostic classification: Clinical (MCI-LB) – clinical diagnosis of MCI-LB; Clinical (DLB) – clinical diagnosis of DLB after conversion to dementia, data reported are from MCI phase.
Articles listed in alphabetical order by first author.

Supplementary Table 3. CASP Checklist for Case-Control Studies

Reference	Q1	Q2	Q3	Q4	Q5	Q6a	Q6b	Q7	Q8	Q9	Q10	Q11
Babiloni 2018 [30]	Y	Y	Y	Y	Y	Y	Y	Y	Y	Y	Y	Y
Belden 2014 [3]	Y	Y	N	N/A	Y	N/A	N/A	N/A	N/A	Y	Y	Y
Blanc 2015 [31]	Y	Y	Y	Y	Y	Y	Y	Y	Y	Y	Y	Y
Blanc 2016 [32]	Y	Y	Y	Y	Y	Y	Y	Y	Y	Y	Y	Y
Bonanni 2015 [4]	Y	Y	Y	Y	Y	Y	Y	Y	Y	Y	Y	Y
Bousiges 2020 [33]	Y	Y	Y	Y	N/A	Y	Y	Y	Y	NC	Y	Y
Bousiges 2016 [34]	Y	Y	Y	Y	N/A	Y	Y	Y	Y	NC	Y	Y
Bousiges 2018 [35]	Y	Y	Y	Y	N/A	Y	Y	Y	Y	NC	Y	Y
Cagnin 2015 [5]	Y	Y	Y	Y	Y	Y	Y	Y	Y	Y	Y	Y
Cagnin 2015 [6]	Y	Y	Y	Y	Y	Y	Y	Y	Y	Y	Y	Y
Caminiti 2019 [7]	Y	Y	Y	Y	Y	NC	Y	Y	Y	Y	Y	Y
Chen 2021 [36]	Y	Y	Y	N/A	Y	N/A	N/A	N/A	N/A	Y	NC	Y
Donaghy 2017 [18]	Y	Y	Y	Y	Y	Y	Y	Y	Y	Y	Y	Y
Donaghy 2018 [19]	Y	Y	Y	Y	Y	Y	Y	Y	Y	Y	Y	Y
Donaghy 2022 [20]	Y	Y	Y	Y	Y	Y	Y	Y	Y	Y	Y	Y
Durcan 2019 [21]	Y	Y	Y	Y	Y	Y	Y	N/A	N/A	Y	NC	NC
Ferman 2013 [8]	Y	Y	Y	Y	Y	Y	N	Y	NC	Y	Y	Y
Firbank 2021 [37]	Y	Y	Y	Y	Y	Y	N	Y	Y	Y	Y	Y
Firbank 2021 [38]	Y	Y	Y	Y	Y	Y	Y	Y	Y	Y	Y	Y
Galvin 2021 [23]	Y	Y	Y	Y	Y	Y	Y	Y	Y	Y	Y	Y
Gan 2022 [22]	Y	Y	Y	Y	Y	Y	Y	Y	Y	Y	Y	Y
Hamilton 2021 [16]	Y	Y	Y	Y	Y	Y	Y	Y	Y	Y	Y	Y
Hamilton 2021 [39]	Y	Y	Y	Y	Y	NC	Y	Y	Y	Y	Y	Y
Hamilton 2021 [15]	Y	Y	Y	Y	Y	Y	Y	Y	Y	Y	Y	Y
Hamilton 2022 [24]	Y	Y	Y	Y	Y	Y	Y	Y	Y	Y	Y	Y
Hamilton 2022b [25]	Y	Y	Y	Y	Y	NC	Y	Y	Y	Y	Y	Y
Iranzo 2014 [40]	Y	Y	Y	N/A	Y	N/A	N/A	N/A	N/A	Y	NC	Y
Jicha 2010 [1]	Y	Y	N	N	Y	Y	Y	Y	Y	Y	Y	Y
Kantarci 2016 [9]	Y	Y	Y	Y	N	Y	Y	Y	Y	Y	Y	Y
Kantarci 2021 [10]	Y	Y	Y	Y	N	Y	Y	Y	Y	Y	Y	Y
Kim 2018 [14]	Y	Y	Y	Y	Y	Y	Y	Y	Y	Y	Y	Y
Kondo 2016 [11]	Y	Y	Y	Y	Y	Y	Y	Y	Y	Y	Y	Y
Liu 2021 [26]	Y	Y	Y	Y	Y	Y	Y	Y	Y	Y	Y	Y
Liu 2022 [27]	Y	Y	Y	Y	Y	Y	Y	Y	Y	Y	Y	Y
Massa 2019 [41]	Y	Y	N	N	Y	Y	Y	Y	Y	Y	Y	Y
Massa 2022 [42]	Y	Y	Y	Y	Y	Y	NC	Y	Y	Y	Y	Y
Molano 2010 [2]	Y	Y	N	N/A	Y	N/A	N/A	N/A	N/A	Y	NC	Y
Payne 2022 [28]	Y	Y	Y	Y	Y	N	Y	Y	Y	Y	Y	Y
Planche 2021 [43]	Y	Y	Y	Y	Y	NC	NC	Y	Y	Y	Y	Y
Postuma 2019 [44]	Y	Y	Y	Y	Y	Y	Y	Y	Y	Y	Y	Y
Roberts 2021 [45]	Y	Y	Y	Y	Y	Y	N	Y	Y	Y	NC	Y
Roberts 2021 [46]	Y	Y	Y	Y	Y	Y	N	Y	NC	Y	NC	NC
Rodriguez Brazète 2013 [47]	Y	Y	Y	Y	Y	Y	Y	Y	Y	Y	Y	Y
Roquet 2016 [48]	Y	Y	Y	Y	Y	Y	Y	Y	Y	Y	Y	Y
Roquet 2017 [49]	Y	Y	Y	Y	Y	Y	Y	Y	Y	Y	Y	Y
Sadiq 2017 [12]	Y	Y	Y	Y	Y	N	N	Y	Y	Y	Y	Y
Schumacher 2020 [50]	Y	Y	Y	Y	Y	Y	Y	Y	Y	Y	Y	Y
Schumacher 2022 [51]	Y	Y	Y	Y	Y	Y	Y	N/A	Y	Y	Y	Y
Siddiqui 2020 [52]	Y	Y	Y	Y	Y	Y	Y	Y	Y	Y	Y	Y
Thomas 2018 [53]	Y	Y	Y	Y	Y	Y	Y	Y	Y	Y	Y	Y
Thomas 2022 [29]	Y	Y	Y	Y	Y	NC	Y	Y	Y	Y	Y	Y
van de Beek 2020 [17]	Y	Y	Y	Y	Y	Y	Y	Y	Y	Y	Y	Y

**van der Zande 2020 [54]**	Y	Y	Y	Y	Y	Y	Y	Y	Y	Y	Y	Y
**Vendette 2012 [55]**	Y	Y	Y	Y	Y	Y	Y	Y	Y	Y	Y	Y
**Yoo 2021 [56]**	Y	Y	N	Y	Y	Y	Y	Y	Y	Y	Y	Y
**Yoon 2015 [13]**	Y	Y	Y	Y	Y	Y	Y	Y	Y	Y	Y	Y
**Yoon 2022 [57]**	Y	Y	Y	Y	Y	Y	Y	Y	Y	Y	Y	Y

Q1: Did the study address a clearly focused issue?

Q2: Did the authors use an appropriate method to answer their question?

Q3: Were cases recruited in an acceptable way? N if number of MCI-LB patients < 15.

Q4: Were controls selected in an acceptable way? N if number of controls/other patients < 15 or control group was insufficiently described.

Q5: Was the exposure accurately measured to minimize bias? N if acquisition parameters not sufficiently described and no reference to previously published methods was made. N/A if not sufficiently described but measure included in meta-analysis was not main outcome of study.

Q6a: Aside from the experimental intervention, were the groups treated equally? This question was interpreted as whether groups were comparable. N if MCI groups were not age-matched. NC if no statistics were reported for comparisons. N/A in the absence of group comparisons.

Q6b: Were potential confounding factors taken into account? N if several demographic factors (age, sex, education) were not matched in Q6a and no correction was applied for any of them. N/A in the absence of group comparisons.

Q7: Can the possibility that the study design affected the effect size be excluded? N/A in the absence of group comparisons.

Q8: How precise was the estimate of the treatment effect? NC if no p-value was provided. N/A in the absence of group comparisons or absence of group differences.

Q9: Do you believe the results? NC if reliability of measures could not be checked.

Q10: Will the results help locally? NC if no significant results were found in the study or data to judge the local applicability was missing in previous columns.

Q11: Do the results fit with other available data? NC if no comparable study

References

[1] Jicha GA, Schmitt FA, Abner E, Nelson PT, Cooper GE, Smith CD, et al. Prodromal clinical manifestations of neuropathologically confirmed Lewy body disease. Neurobiology of aging. 2010;31:1805-13.
[2] Molano J, Boeve B, Ferman T, Smith G, Parisi J, Dickson D, et al. Mild cognitive impairment associated with limbic and neocortical Lewy body disease: a clinicopathological study. Brain : a journal of neurology. 2010;133:540-56.
[3] Belden CM, Kahlon V, Malek-Ahmadi M, Tsai A, Sabbagh MN. Clinical characterization of mild cognitive impairment as a prodrome to dementia with lewy bodies. American Journal of Alzheimer's Disease and other Dementias. 2014;30:173-7.
[4] Bonanni L, Perfetti B, Bifolchetti S, Taylor JP, Franciotti R, Parnetti L, et al. Quantitative electroencephalogram utility in predicting conversion of mild cognitive impairment to dementia with Lewy bodies. Neurobiology of aging. 2015;36:434-45.
[5] Cagnin A, Busse C, Gardini S, Jelcic N, Guzzo C, Gnoato F, et al. Clinical and Cognitive Phenotype of Mild Cognitive Impairment Evolving to Dementia with Lewy Bodies. Dement Geriatr Cogn Dis Extra. 2015;5:442-9.
[6] Cagnin A, Busse C, Jelcic N, Gnoato F, Mitolo M, Caffarra P. High specificity of MMSE pentagon scoring for diagnosis of prodromal dementia with Lewy bodies. Parkinsonism & related disorders. 2015;21:303-5.
[7] Caminiti SP, Sala A, Iaccarino L, Beretta L, Pilotto A, Gianolli L, et al. Brain glucose metabolism in Lewy body dementia: implications for diagnostic criteria. Alzheimer's research & therapy. 2019;11:20.
[8] Ferman TJ, Smith GE, Kantarci K, Boeve BF, Pankratz VS, Dickson DW, et al. Nonamnestic mild cognitive impairment progresses to dementia with Lewy bodies. Neurology. 2013;81:2032-8.
[9] Kantarci K, Lesnick T, Ferman TJ, Przybelski SA, Boeve BF, Smith GE, et al. Hippocampal volumes predict risk of dementia with Lewy bodies in mild cognitive impairment. Neurology. 2016;87:2317-23.
[10] Kantarci K, Boeve BF, Przybelski SA, Lesnick TG, Chen Q, Fields J, et al. FDG PET metabolic signatures distinguishing prodromal DLB and prodromal AD. NeuroImage: Clinical. 2021;31.
[11] Kondo D, Ota K, Kasanuki K, Fujishiro H, Chiba Y, Murayama N, et al. Characteristics of mild cognitive impairment tending to convert into Alzheimer's disease or dementia with Lewy bodies: A follow-up study in a memory clinic. Journal of the neurological sciences. 2016;369:102-8.
[12] Sadiq D, Whitfield T, Lee L, Stevens T, Costafreda S, Walker Z. Prodromal Dementia with Lewy Bodies and Prodromal Alzheimer's Disease: A Comparison of the Cognitive and Clinical Profiles. Journal of Alzheimer's disease : JAD. 2017;58:463-70.
[13] Yoon JH, Kim M, Moon SY, Yong SW, Hong JM. Olfactory function and neuropsychological profile to differentiate dementia with Lewy bodies from Alzheimer's disease in patients with mild cognitive impairment: A 5-year follow-up study. Journal of the neurological sciences. 2015;355:174-9.
[14] Kim MS, Yoon JH, Hong JM. Early differentiation of dementia with Lewy bodies and Alzheimer's disease: Heart rate variability at mild cognitive impairment stage. Clinical Neurophysiology. 2018;129:1570-8.
[15] Hamilton CA, Matthews FE, Donaghy PC, Taylor JP, O'Brien JT, Barnett N, et al. Progression to Dementia in Mild Cognitive Impairment With Lewy Bodies or Alzheimer Disease. Neurology. 2021.
[16] Hamilton CA, Matthews FE, Donaghy PC, Taylor JP, O'Brien JT, Barnett N, et al. Cognitive Decline in Mild Cognitive Impairment With Lewy Bodies or Alzheimer Disease: A Prospective Cohort Study. The American journal of geriatric psychiatry : official journal of the American Association for Geriatric Psychiatry. 2021;29:272-84.
[17] van de Beek M, van Steenoven I, van der Zande JJ, Barkhof F, Teunissen CE, van der Flier WM, et al. Prodromal Dementia With Lewy Bodies: Clinical Characterization and Predictors of Progression. Movement disorders : official journal of the Movement Disorder Society. 2020;35:859-67.

[18] Donaghy PC, Barnett N, Olsen K, Taylor JP, McKeith IG, O'Brien JT, et al. Symptoms associated with Lewy body disease in mild cognitive impairment. International journal of geriatric psychiatry. 2017;32:1163-71.
[19] Donaghy PC, Taylor JP, O'Brien JT, Barnett N, Olsen K, Colloby SJ, et al. Neuropsychiatric symptoms and cognitive profile in mild cognitive impairment with Lewy bodies. Psychological medicine. 2018;48:2384-90.
[20] Donaghy PC, Ciafone J, Durcan R, Hamilton CA, Barker S, Lloyd J, et al. Mild cognitive impairment with Lewy bodies: neuropsychiatric supportive symptoms and cognitive profile. Psychological medicine. 2022;52:1147-55.
[21] Durcan R, Donaghy PC, Barnett NA, Olsen K, Yarnall AJ, Taylor JP, et al. Prevalence and severity of symptoms suggestive of gastroparesis in prodromal dementia with Lewy bodies. International journal of geriatric psychiatry. 2019;34:990-8.
[22] Gan J, Liu S, Chen Z, Yang Y, Ma L, Meng Q, et al. Elevated Plasma Orexin-A Levels in Prodromal Dementia with Lewy Bodies. Journal of Alzheimer's disease : JAD. 2022;88:1037-48.
[23] Galvin JE, Chrisphonte S, Cohen I, Greenfield KK, Kleiman MJ, Moore C, et al. Characterization of dementia with Lewy bodies (DLB) and mild cognitive impairment using the Lewy body dementia module (LBD-MOD). Alzheimer's and Dementia. 2021;17:1675-86.
[24] Hamilton CA, Frith J, Donaghy PC, Barker SAH, Durcan R, Lawley S, et al. Assessment of autonomic symptoms may assist with early identification of mild cognitive impairment with Lewy bodies. International journal of geriatric psychiatry. 2022;37.
[25] Hamilton CA, Frith J, Donaghy PC, Barker SAH, Durcan R, Lawley S, et al. Blood pressure and heart rate responses to orthostatic challenge and Valsalva manoeuvre in mild cognitive impairment with Lewy bodies. International journal of geriatric psychiatry. 2022;37.
[26] Liu C, Liu S, Wang X, Ji Y. Neuropsychiatric profiles in mild cognitive impairment with Lewy bodies. Aging & mental health. 2021;25:2011-7.
[27] Liu S, Liu C, Hu W, Ji Y. Frequency, Severity, and Duration of Autonomic Symptoms in Patients of Prodromal Dementia with Lewy Bodies. Journal of Alzheimer's disease : JAD. 2022;89:923-9.
[28] Payne S, Shofer JB, Shutes-David A, Li G, Jankowski A, Dean P, et al. Correlates of Conversion from Mild Cognitive Impairment to Dementia with Lewy Bodies: Data from the National Alzheimer's Coordinating Center. Journal of Alzheimer's disease : JAD. 2022;86:1643-54.
[29] Thomas AJ, Hamilton CA, Barker S, Durcan R, Lawley S, Barnett N, et al. Olfactory impairment in mild cognitive impairment with Lewy bodies and Alzheimer's disease. International psychogeriatrics / IPA. 2022;34:585-92.
[30] Babiloni C, Del Percio C, Lizio R, Noce G, Lopez S, Soricelli A, et al. Abnormalities of Resting State Cortical EEG Rhythms in Subjects with Mild Cognitive Impairment Due to Alzheimer's and Lewy Body Diseases. Journal of Alzheimer's disease : JAD. 2018;62:247-68.
[31] Blanc F, Colloby SJ, Philippi N, de Petigny X, Jung B, Demuynck C, et al. Cortical Thickness in Dementia with Lewy Bodies and Alzheimer's Disease: A Comparison of Prodromal and Dementia Stages. PloS one. 2015;10:e0127396.
[32] Blanc F, Colloby SJ, Cretin B, de Sousa PL, Demuynck C, O'Brien JT, et al. Grey matter atrophy in prodromal stage of dementia with Lewy bodies and Alzheimer's disease. Alzheimer's research & therapy. 2016;8:31.
[33] Bousiges O, Philippi N, Lavaux T, Perret-Liaudet A, Lachmann I, Schaeffer-Agalede C, et al. Differential diagnostic value of total alpha-synuclein assay in the cerebrospinal fluid between Alzheimer's disease and dementia with Lewy bodies from the prodromal stage. Alzheimer's Research and Therapy. 2020;12.
[34] Bousiges O, Cretin B, Lavaux T, Philippi N, Jung B, Hezard S, et al. Diagnostic Value of Cerebrospinal Fluid Biomarkers (Phospho-Tau181, total-Tau, Abeta42, and Abeta40) in Prodromal Stage of Alzheimer's Disease and Dementia with Lewy Bodies. Journal of Alzheimer's disease : JAD. 2016;51:1069-83.

[35] Bousiges O, Bombois S, Schraen S, Wallon D, Quillard MM, Gabelle A, et al. Cerebrospinal fluid Alzheimer biomarkers can be useful for discriminating dementia with Lewy bodies from Alzheimer's disease at the prodromal stage. Journal of neurology, neurosurgery, and psychiatry. 2018;89:467-75.
[36] Chen Q, Lowe VJ, Boeve BF, Przybelski SA, Miyagawa T, Senjem ML, et al. Mild Cognitive Impairment at Risk for Lewy Body Dementia. Neurology. 2021;6.
[37] Firbank MJ, O'Brien JT, Durcan R, Allan LM, Barker S, Ciafone J, et al. Mild cognitive impairment with Lewy bodies: blood perfusion with arterial spin labelling. J Neurol. 2021;268:1284-94.
[38] Firbank MJ, Durcan R, O'Brien JT, Allan LM, Barker S, Ciafone J, et al. Hippocampal and insula volume in mild cognitive impairment with Lewy bodies. Parkinsonism & related disorders. 2021;86:27-33.
[39] Hamilton CA, Schumacher J, Matthews F, Taylor JP, Allan L, Barnett N, et al. Slowing on quantitative EEG is associated with transition to dementia in mild cognitive impairment. International psychogeriatrics / IPA. 2021;33:1321-5.
[40] Iranzo A, Fern, ez-Arcos A, Tolosa E, Serradell M, Molinuevo JL, et al. Neurodegenerative disorder risk in idiopathic REM sleep behavior disorder: Study in 174 patients. PloS one. 2014;9.
[41] Massa F, Arnaldi D, De Cesari F, Girtler N, Brugnolo A, Grazzini M, et al. Neuroimaging findings and clinical trajectories of Lewy body disease in patients with MCI. Neurobiology of aging. 2019;76:9-17.
[42] Massa F, Chincarini A, Bauckneht M, Raffa S, Peira E, Arnaldi D, et al. Added value of semiquantitative analysis of brain FDG-PET for the differentiation between MCI-Lewy bodies and MCI due to Alzheimer's disease. European journal of nuclear medicine and molecular imaging. 2022;49:1263-74.
[43] Planche V, Bouteloup V, Mangin JF, Dubois B, Delrieu J, Pasquier F, et al. Clinical relevance of brain atrophy subtypes categorization in memory clinics. Alzheimer's and Dementia. 2021;17:641-52.
[44] Postuma RB, Iranzo A, Hu M, Hogl B, Boeve BF, Manni R, et al. Risk and predictors of dementia and parkinsonism in idiopathic REM sleep behaviour disorder: a multicentre study. Brain : a journal of neurology. 2019;142:744-59.
[45] Roberts G, Donaghy PC, Lloyd J, Durcan R, Petrides G, Colloby SJ, et al. Accuracy of dopaminergic imaging as a biomarker for mild cognitive impairment with Lewy bodies. The British journal of psychiatry : the journal of mental science. 2021;218:276-82.
[46] Roberts G, Durcan R, Donaghy PC, Lawley S, Ciafone J, Hamilton CA, et al. Accuracy of Cardiac Innervation Scintigraphy for Mild Cognitive Impairment With Lewy Bodies. Neurology. 2021;96:e2801-e11.
[47] Rodrigues Brazète J, Montplaisir J, Petit D, Postuma RB, Bertr, JA, et al. Electroencephalogram slowing in rapid eye movement sleep behavior disorder is associated with mild cognitive impairment. Sleep medicine. 2013;14:1059-63.
[48] Roquet D, Sourty M, Botzung A, Armspach JP, Blanc F. Brain perfusion in dementia with Lewy bodies and Alzheimer's disease: An arterial spin labeling MRI study on prodromal and mild dementia stages. Alzheimer's Research and Therapy. 2016;8.
[49] Roquet D, Noblet V, Anthony P, Philippi N, Demuynck C, Cretin B, et al. Insular atrophy at the prodromal stage of dementia with Lewy bodies: a VBM DARTEL study. Scientific reports. 2017;7:9437.
[50] Schumacher J, Taylor JP, Hamilton CA, Firbank M, Cromarty RA, Donaghy PC, et al. Quantitative EEG as a biomarker in mild cognitive impairment with Lewy bodies. Alzheimer's research & therapy. 2020;12:82.
[51] Schumacher J, Taylor JP, Hamilton CA, Firbank M, Donaghy PC, Roberts G, et al. Functional connectivity in mild cognitive impairment with Lewy bodies. J Neurol. 2021;268:4707-20.
[52] Siddiqui TG, Whitfield T, Praharaju SJ, Sadiq D, Kazmi H, Ben-Joseph A, et al. Magnetic Resonance Imaging in Stable Mild Cognitive Impairment, Prodromal Alzheimer's Disease, and Prodromal Dementia with Lewy Bodies. Dementia and geriatric cognitive disorders. 2020;49:583-8.

[53] Thomas AJ, Donaghy P, Roberts G, Colloby SJ, Barnett NA, Petrides G, et al. Diagnostic accuracy of dopaminergic imaging in prodromal dementia with Lewy bodies. Psychological medicine. 2018:1-7.
[54] van der Zande JJ, Gouw AA, van Steenoven I, van de Beek M, Scheltens P, Stam CJ, et al. Diagnostic and prognostic value of EEG in prodromal dementia with Lewy bodies. Neurology. 2020;95:e662-e70.
[55] Vendette M, Montplaisir J, Gosselin N, Soucy J-P, Postuma RB, Dang-Vu TT, et al. Brain perfusion anomalies in rapid eye movement sleep behavior disorder with mild cognitive impairment. Movement disorders : official journal of the Movement Disorder Society. 2012;27:1255-61.
[56] Yoo D, Lee JY, Kim YK, Yoon EJ, Kim H, Kim R, et al. Mild cognitive impairment and abnormal brain metabolic expression in idiopathic REM sleep behavior disorder. Parkinsonism & related disorders. 2021;90:1-7.
[57] Yoon EJ, Lee JY, Kim H, Yoo D, Shin JH, Nam H, et al. Brain Metabolism Related to Mild Cognitive Impairment and Phenoconversion in Patients With Isolated REM Sleep Behavior Disorder. Neurology. 2022;98:e2413-e24.
